# Social Media Use for Research Participant Recruitment: Integrative Literature Review

**DOI:** 10.2196/38015

**Published:** 2022-08-04

**Authors:** Elizabeth Mirekuwaa Darko, Manal Kleib, Joanne Olson

**Affiliations:** 1 College of Health Sciences, Faculty of Nursing Edmonton Clinic Health Academy University of Alberta Edmonton, AB Canada

**Keywords:** advertisement, recruitment, research participants, social media, mobile phone

## Abstract

**Background:**

Social media tools have provided health researchers with the opportunity to engage with communities and groups in a nonconventional manner to recruit participants for health research. Using social media to advertise research opportunities and recruit participants facilitates accessibility to participants from broad geographical areas and diverse populations. However, little guidance is provided by ethics review boards for researchers to effectively use this recruitment method in their research.

**Objective:**

This study sought to explore the literature on the use of social media for participant recruitment for research studies and identify the best practices for recruiting participants using this method.

**Methods:**

An integrative review approach was used to synthesize the literature. A total of 5 health sciences databases, namely, EMBASE (Ovid), MEDLINE (Ovid and EBSCOhost), PsycINFO (Ovid), Scopus (Elsevier), and CINAHL Plus with Full Text (EBSCOhost), were searched using predefined keywords and inclusion and exclusion criteria. The initial search was conducted in October 2020 and was updated in February 2022. Descriptive and content analyses were applied to synthesize the results, and the findings are presented in a narrative and tabular format.

**Results:**

A total of 96 records were included in this review, 83 (86%) from the initial search and 13 (14%) from the updated search. The publication year ranged between 2011 and 2022, with most publications (63/96, 66%) being from the United States. Regarding recruitment strategy, 45% (43/96) of the studies exclusively used social media, whereas 51% (49/96) used social media in conjunction with other strategies. The remaining 4% (4/96) provided guidelines and recommendations for social media recruitment. Notably, 38% (36/96) of these studies involved hard-to-reach populations. The findings also revealed that the use of social media is a cost-effective and efficient strategy for recruiting research participants. Despite the expanded use across different populations, there is limited participation of older adults in social media recruitment.

**Conclusions:**

This review provides important insights into the current use of social media for health research participant recruitment. Ethics boards and research support services in academic institutions are encouraged to explicitly provide researchers with guidelines on the use of social media for health research participant recruitment. A preliminary guideline prepared based on the findings of this review is proposed to spark further development in this area.

## Introduction

### Background

In this digital age, advancements in technology have created opportunities for researchers to use new techniques to recruit research participants. For health researchers, technological innovations present an opportunity to use digital platforms such as social media, the internet, web applications, multimedia, and smartphones to effectively and efficiently engage the community for research recruitment [1]. These digital platforms provide an additional source for participant recruitment for health research studies [2]. Within health sciences, social media is being quickly adopted because of its increased use as a method of communication with the public [3]. For many researchers, recruiting participants for trials can be a daunting task that can result in study delays or the termination of trials [4]. Less than one-third of trials reach their original target within a specified time frame, and approximately one-third required extension [5]. Hence, reaching targeted participants through social media platforms provides an important avenue for facilitating researchers’ work.

Social media refers to a group of internet-based communication services through which users create and participate in web-based exchanges, contribute user-created content such as videos, or join web-based communities to share information and ideas [6]. The trends and patterns of social engagement worldwide help provide researchers, policy makers, and other stakeholders with an overview of the different social media applications that users are engaged with [7] and how these tools could potentially be used to leverage health research. With a global population of 7.8 billion inhabitants [8], internet users stand at 4.54 billion, representing a 59% penetration rate, and active social media users at 3.80 billion, representing 49% [9,10]. Active social media platforms users include Facebook (63%), YouTube (61%), WhatsApp (48%), Facebook Messenger (38%), Instagram (36%), Twitter (23%), and Snapchat (13%) [9,11]. Social media provides an appropriate medium for user connection and communication, information collection and dissemination, knowledge sharing, discussion, and collaboration with communities for professional networking and business purposes [12-14].

Despite the numerous benefits and opportunities associated with social media, its use in the recruitment of research participants is still evolving. Health researchers using digital platforms for research participant recruitment encounter challenges such as efficiency, cost, information reliability, informed consent, confidentiality, privacy-related concerns [15], internet accessibility, information overload, informed consent, and interaction quality [12,13]. In traditional recruitment methods, researchers often face costs associated with personnel and resources, administrative changes, time-consuming recruitment processes, recruitment bias, and population homogeneity [16-20]. Cost plays an essential part in the success of a research process as a higher fraction of the cost is allocated to participant recruitment [21]. The cost involved in research studies varies and is dependent on certain factors such as the targeted population, geographical location, and type of recruitment approach [18]. To overcome the challenges associated with the cost of participant recruitment, researchers need metrics to determine the cost of recruitment.

To access social media, users are required to create a profile that requires certain mandatory information such as first name and last name, email address, or mobile phone number [22]. Although interested social media users willingly provide these data, they are often unknowingly signing away their privacy, which increases the possibility of privacy breaches [23]. Although research ethics boards (REBs) require removing identifying information of research participants from data using unique identifiers, such guidelines are rendered ineffective in the context of social media data as participants’ relational links are predictive of their attributes [24]. Nonetheless, Narayanan and Shmatikov [25] stated that such anonymization of participants’ data might be insufficient to protect social media networks’ privacy.

Researchers need guidance to navigate the ethical and logistical issues associated with using social platforms as a recruitment tool other than the “Terms and Conditions” stated by the application software providers [26]. Therefore, researchers often turn to ethics boards within their institutions for guidance on social media and internet recruitment; however, this information is not always readily available. To determine this, we reviewed the REBs of the top 10 higher education institutions in Canada to identify any standard ethical guidelines currently being used or recommended for using social media tools to recruit participants for research studies beyond adopting the Tri-Council Policy Statement on research. We used the QS World University Ranking criteria, which determine universities’ rankings worldwide based on 6 metrics [27]. This strategy was deemed appropriate as these universities are known for their high-impact research productivity. The results revealed that only 3 universities had guidelines available on social media use in research studies, which further supported the need for this integrative review (Multimedia Appendix 1). A standard protocol that could be adopted by postsecondary institutions, research organizations, and researchers could help mitigate the pitfalls researchers encounter during participant recruitment for research via social media applications. Such protocols may facilitate the research process, expedite data collection, and ensure that digital research recruitment practices protect participants’ data and rights.

Regarding the published literature, only 1 review [15] examined the evidence of cost, effectiveness, and the characteristics of participants recruited through Facebook compared with other web-based, social media, and traditional recruitment methods for adult health research. Little is known about the use of other social media platforms for participant recruitment in health research. Therefore, this study was warranted to address these gaps in the literature.

### Objectives and Research Questions

This review sought to examine the evidence available on all the applications identified as social media tools and identify the best practices to facilitate participant recruitment through these tools. We addressed the following research questions:

1. What are the different social media tools commonly used by health science researchers for recruiting research participants and in what populations?
2. What is the proportion of nursing researchers who use social media platforms for recruitment?
3. What are the benefits and challenges of using social media to recruit research participants?
4. What are the best practices and ethical considerations for using social media tools to recruit research participants?

## Methods

### Overview

An integrative review guided by the Whittemore and Knafl [28] framework was conducted. This review type allows for the inclusion of diverse research methodologies and data sources to understand and generate new knowledge on the phenomenon of interest [28,29]. A comprehensive search strategy was formulated in consultation with a health science librarian. The initial search was conducted on October 11, 2020, and updated on February 24, 2022, in the EMBASE (Ovid), MEDLINE (Ovid and EBSCOhost), PsycINFO (Ovid), Scopus (Elsevier), and CINAHL Plus with Full Text (EBSCOhost) databases using a search strategy of keywords and subject headings through an iterative process (Multimedia Appendix 2). The criteria for eligibility were (1) all types of published research on primary and secondary studies, including qualitative, quantitative, and mixed methods; (2) discussion papers, white papers, reports, brief reports, specific guidelines, conference proceedings, dissertations, and published manuscripts that reported on social media use; (3) research reports published between January 2000 and February 2022; and (4) research reports that focused on research participant recruitment and advertisements on social media platforms, including all types of populations and health sciences disciplines, and (5) all geographical locations. The following articles were excluded: (1) non–English-language articles; (2) unpublished manuscripts and non–peer-reviewed publications such as descriptive papers, editorial papers, opinion papers, letters, book reviews, and article reviews; (3) review articles (scoping, integrative, narrative, and systematic) already published on the topic; and (4) all non–health sciences articles. The time frame for the published reports was chosen to capture the contemporary views that reflect the trends and popularity of digital platforms in participant recruitment.

### Data Evaluation and Analysis

**Figure 1:**
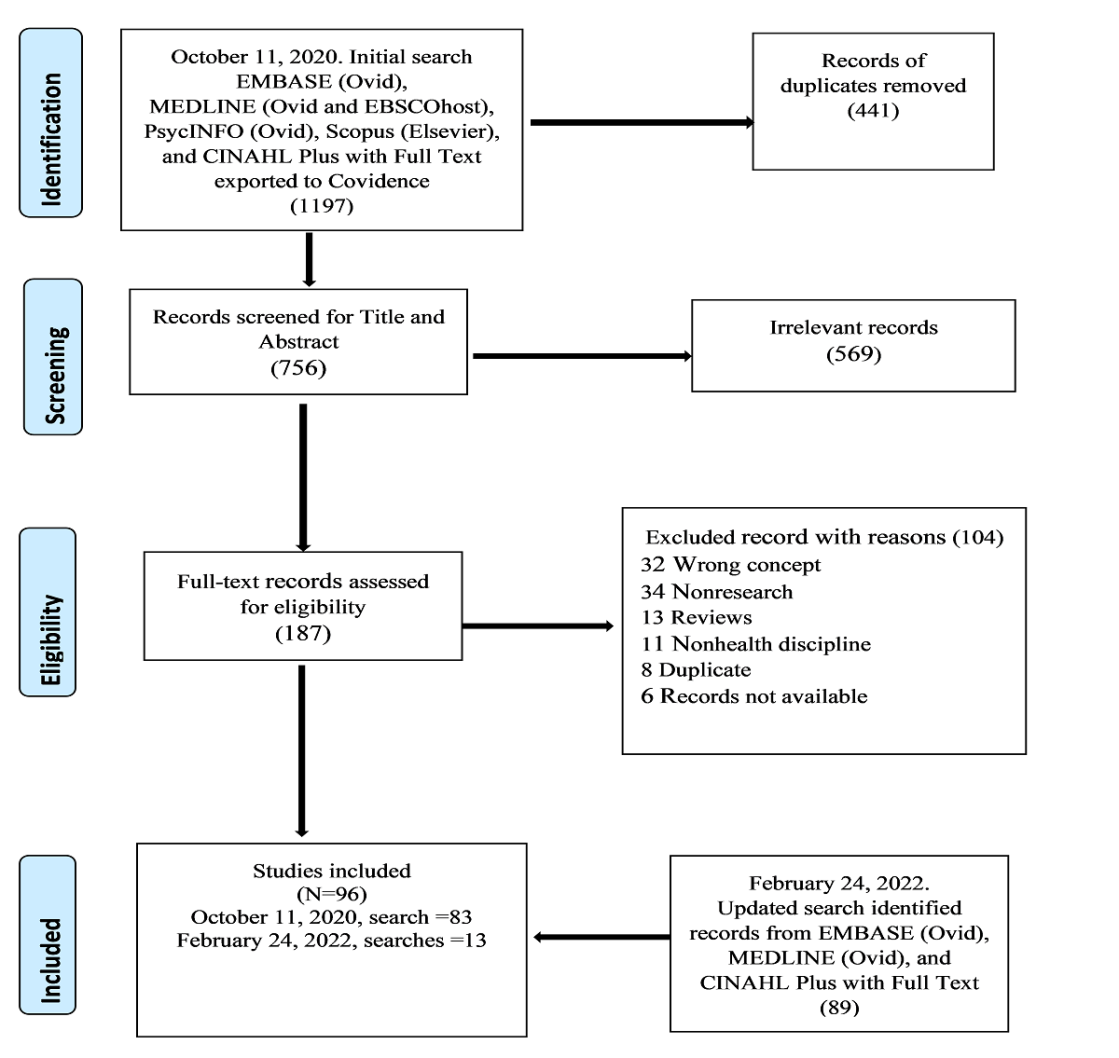
PRISMA (Preferred Reporting Items for Systematic Reviews and Meta-Analyses) 2009 flow diagram.

Records from the databases (initial search N=1197) were retrieved and imported into the Covidence Management Software for data screening and extraction. Overall, 2 reviewers (EMD and MK) independently conducted the screening process in Covidence, screening titles and abstracts, followed by full-text screening. All decisions made to either include or exclude records against the predetermined inclusion and exclusion criteria were documented. Where conflicts arose, the 2 reviewers consulted and resolved them through a voting process. We conducted another search on February 24, 2022, to update the results. The PRISMA (Preferred Reporting Items for Systematic Reviews and Meta-Analyses) Protocols template in Covidence was used to map out the screening process, and the results as shown in Figure 1.

The following details were extracted from the included records: name or names of the author or authors, year of publication, country of publication, study design, study population, total number recruited, total number of participants enrolled or recruited through social media, social media platform used, other recruitment strategies, type of advertisement (paid or not paid), incentives provided, whether the study was funded, limitations of social media reported by the authors, and duration of advertisement (Multimedia Appendix 3 [2,18,20,30-115]). The extracted data from these records were analyzed by identifying codes and categories to characterize emerging themes, patterns, trends, and relationships to aid in synthesizing the findings logically and coherently. In addition, descriptive statistics were applied where appropriate to describe and summarize the data pertinent to the distribution of research and other characteristics. The Critical Appraisal Skills Program [116] was used per the research methodology to appraise and evaluate each of the included studies critically to ensure the quality of the available evidence included in this review (Multimedia Appendix 4 [18,20,31,50,53,54,55,58,60,61,76,77,81,106,109,114]). The studies were assessed and rated as “low quality” or “moderate quality” based on their theoretical or methodological rigor [28]. Ethics approval was not required as this study did not involve human participants.

## Results

### Overview

In total, 1197 records were retrieved from the initial database search; in Covidence, 441 (36.8%) duplicate records were removed from the imported references, and a total of 756 (63.2%) records were moved for screening. In the first stage of screening, the titles and abstracts of each record were screened for full-text review. In the second stage of screening, 187 full-text studies were reviewed entirely and assessed for eligibility for inclusion or exclusion. For records that were not available in the full text, the Health Science Library was contacted to obtain those records. A total of 6 articles that reported on conference proceedings were retrieved but did not have any substantial information, as reported in the abstracts. Nonetheless, the full texts of these articles were requested through library services but could not be retrieved; thus, a decision was made to exclude them from the results. It should be noted that although the Covidence software automatically removes duplicates, there were instances of errors where some records were missed; therefore, removing these duplicates manually was warranted. Of the initial search, a total of 83 records were included. The updated search returned a total of 89 records. Of the 89 articles, after 11 (12%) duplicates were removed, a total of 78 (88%) articles were screened. A total of 23 articles underwent full-text review, of which 10 (43%) were excluded as they did not meet the inclusion criteria, and 13 (57%) articles were retained and included in the review. Finally, 96 records were included in the review. In total, 114 records were excluded (Multimedia Appendix 5).

### Characteristics of Included Studies

The range of publication years of the articles included in the review was between 2011 and 2022 (Multimedia Appendix 3). Most publications were from the United States (63/96, 66%) and Australia (20/96, 21%). Besides that, they were from Canada (5/96, 5%), the Netherlands (2/96, 2%), and the United Kingdom (2/96, 2%). There was only one publication from each of the following countries: Taiwan, Ecuador, India, and Brazil. Out of the 96 included studies, 92 (96%) were papers reporting primary research and 4 (4%) were reports on using social media. The methodological approaches used were cross-sectional studies (38/96, 40%), web-based surveys (15/96, 16%), secondary data analysis (14/96, 15%), randomized controlled trials (10/96, 10%), reports (4/96, 4%), mixed methods studies (4/96, 4%), qualitative studies (3/96, 3%), cohort studies (3/96, 3%), clinical trials (3/96, 3%), quasi-experimental studies (1/96, 1%), and longitudinal studies (1/96, 1%).

### Social Media Use by Nursing Researchers and Other Health Researchers

We were interested in determining the proportion of nursing researchers using social media; however, this was not easy to identify as researchers have published studies that have used social media for the recruitment of research participants in a variety of interdisciplinary journals. On the basis of the journal names where these articles were published, these researchers could be from any health discipline, including nursing, medicine, psychology, rehabilitation, nutrition, pharmacy, or public health. Of the 96 included studies, 71 (74%) were published in general health science journals or interdisciplinary journals [18,20,26,30-92,117-121], and 25 (26%) were published in nursing-related journals [2,93-115,122].

### Social Media Tools Commonly Used by Health Researchers

Researchers used a variety of social media platforms to recruit participants, as reported in the included studies. Researchers either exclusively used social media (43/96, 45%) or social media in conjunction with other recruitment methods (49/96, 51%) to recruit participants (Multimedia Appendix 3). For studies that exclusively used social media, ≥1 social media platform was used simultaneously. Social media platforms included Facebook, Twitter, Craigslist, Instagram, YouTube, LinkedIn, Reddit, Snapchat, and Tumblr. For social media in conjunction with other recruitment strategies, researchers used the identified social media platforms in addition to blogs, social media, Grindr, and WhatsApp Messenger. It was noted that, at times, researchers used the term “social media” but did not specify the type of social media used. In both approaches, most participants relied on the use of Facebook for research recruitment.

### Age Groups of Research Participants Recruited via Social Media

Although researchers used social media platforms for advertisement and recruitment of participants for research, they sometimes did not target specific populations. In addition, the age group distribution of these populations varied, and the definition of the age group differed depending on the study aims. To address this, initially, the range of age groups was specified as follows: children (aged <9 years), adolescents (aged 10-18 years), young adults (aged 19-35 years), middle-aged adults (aged 36-55 years), older adults (aged 56-64 years), and older adults aged ≥65 years. The included studies were then scanned against this categorization to identify which age group was most targeted for social media recruitment (Table 1).

**Table 1:** Age groups of research participants recruited via social media (N=92).

Age category	Participants, n (%)	Research studies
≥18 years	28 (30)	[30,31,33-35,40,48,50,52,57,58,70,73-76,78,79,81-83,90,94,97,108,111,112,114,115]
Age group: not specified	22 (24)	[2,32,36,39,56,66,68,86,89,92,93,96,98-101,103,105-107,110,119]
Two age categories	18 (20)	[20,38,41,42,46,47,49,53,55,58,61,63,64,72,84,87,95,102]
Age groups between 3 age categories	9 (10)	[37,44,54,62,67,77,80,85,117]
Age groups between 4 age categories	3 (3)	[60,71,91]
Age groups between 5 age categories	3 (3)	[65,69,113]
Adolescents	2 (2)	[18,120]
Age groups of ≥21 years	3 (3)	[52,88,109]
≥30 years	1 (1)	[51]
≥33 years	1 (1)	[104]
≥45 years	1 (1)	[45]
≥60 years	1 (1)	[43]
Age group of ≤9 years	0 (0)	—^a^

^a^Not available.

### Populations Targeted in Social Media Recruitment and Their Characteristics

Researchers have targeted different populations in their research studies. Largely, there were many studies (46/96, 48%) that included the general population [2,18,20,31,32, 36,37,40,41, 43,46,47,49, 51,54,55, 58,60,63,64,65, 67,69,70,71,72,73,74,77, 79,80,84, 85,91,92,93,94, 104,106,108, 112,114,115, 119,120]. In addition, a significant proportion (36/96, 38%) of the included studies focused on recruiting hard-to-reach populations (Table 2). Hard-to-reach populations are groups that are socially disadvantaged and present a challenge to access for researchers because of ethnicity, low income, or health literacy [123,124]. In this review, these populations had addiction problems, unique medical disease conditions, or lifestyle choices or belonged to an ethnic minority group. A few studies applied social media recruitment to target health care professionals as research participants (10/96, 10%) [96,98,99,100,101,103,105,106,107,113].

**Table 2:** Hard-to-reach populations targeted in social media recruitment.

Population	Records
Addiction: smoking and alcohol	[30,35,37,38,44,48,50,52,81]
Medical disease conditions: survivor of cancer, autism spectrum disorder, Lynch syndrome, people living with HIV, asthma, obstructive pulmonary disease, depression, and kidney transplant recipient	[34,39,42,53,56,58,61,66,68,89,97,107,111]
Lifestyle: men who have sex with men	[33,57,78,82,83,90]
Ethnic minorities: low-income and racial minority	[45,62,75,76,86,88,95,102]

### Cost-effectiveness, Efficacy, and Feasibility of Social Media in Comparison With Other Recruitment Methods

The costs of recruitment reported in all the included studies are presented in tabular form (Tables 3 and 4) to help ascertain how money was dispensed, as well as the cost-effectiveness of each recruitment strategy. However, it was noted that although some researchers included personnel costs, advertising costs, and other recruitment costs in the total cost, other researchers did not include these costs. Hence, researchers should use only this information as a guide. Few studies specifically compared social media effectiveness to other platforms with the goal of establishing cost-effectiveness, efficacy, and feasibility. In this review, 7% (7/96) of the included studies aimed to determine the effectiveness of social media compared with other recruitment strategies [39,51,52,58,70,74,106], and 9% (9/96) of studies that did not compare social media platforms with any other strategy [30,38,41,46,63,72,73,93,111] found social media as an effective recruitment strategy in both instances.

Few studies did not conclusively find social media to be cost-effective or efficient. The findings reported by these researchers differ because of the different populations targeted, scale of recruitment, and whether the research was funded. In their funded study, Moreno et al [18] found that in-person strategies yielded more participants in a geographic area at a lower cost than social media, and the cost per enrollee by social media was higher than that of traditional methods. In addition, Frandsen et al [48] suggested that Facebook was cost-effective in obtaining eligible participants at the initial stage of the recruitment process. The mailing of letters was cost-effective compared with Facebook, according to Waltman et al [106].

**Table 3:** Cost of social media recruitment methods.

Study and social media			Cost per person		Total cost
**Ahmed et al [39]**					
	Facebook	US $8.73		US $2950	
**Wilkerson et al [82]**					
	Social media advertisements, posts, and email blasts	US $40.44		US $3033.11	
	Social media posts and website banner advertisements	US $15.86		US $1380	
	Social media posts and film festival entrance wavier	US $2.78		US $6297.66	
**Gioia et al [52]**					
	Craigslist	US $1.46		US $275	
**Musiat et al [64]**					
	Facebook	Aus $105.77 (US $73.07)		—^a^	
	Twitter	Aus $422.03 (US $292.08)		—	
	YouTube	Aus $81.31 (US $56.27)		—	
**Frandsen et al [50]**					
	Facebook	Aus $42.34 (US $29.30)		Aus $5842.30 (US $4043.35)	
**Byaruhanga et al [35]**					
	Facebook	Aus $61.68 (US $42.69)		Aus $33,738.52 (US $23349.83)	
	Twitter	Aus $61.52 (US $42.58)		Aus $61.52 (US $42.58)	
**Harris et al [55]**					
	Facebook	—		Aus $28,571.54 (US $19,773.86)	
**Moreno et al [18]**					
	Social media (Facebook, Twitter, and blogs)	US $40.99		—	
**Wilkerson et al [83]**					
	Facebook and Twitter	—		Free	
**Guthrie et al [54]**					
	Facebook	—		US $14,825	
**Waltman et al [106]**					
	Facebook	US $119.38		US $5252.83	
**Watson et al [81]**					
	Facebook	US $40.51 (randomized)		US $49,791.49	
**Derrick et al [44]**					
	Facebook	US $498 per couple		US $10,966	
	Facebook	US $181 per couple		US $4145	
**Carter-Harris et al [117]**					
	Facebook	—		US $500	
**Jones et al [95]**					
	Facebook	US $66.46 (randomized)		—	
**Frandsen et al [48]**					
	Facebook	Aus $56.34 (US $38.99)		Aus $5183.33 (US $3587.29)	
**Juraschek et al [58]**					
	Facebook	US $794		US $5704	
	Facebook	US $1426		US $2383	
**Iott et al [57]**					
	Grindr	US $87.35		US $1747.40	
	Scruff	US $69.30		US $207.90	
	Facebook	US $149.90		US $170.69	
	Facebook groups	—		US $10.40	
**van Gelder et al [77]**					
	Facebook	€10.88 (US $11.13)		€315.52 (US $322.89)	
	Facebook	€9.48 (US $9.70)		€284.48 (US $291.13)	
**Alley et al [70]**					
	Untargeted Facebook	Aus $68 (US $47.06)		Aus $1438 (US $995.21)	
	Targeted Facebook	Aus $42 (US $29.07)		Aus $7721(US $5343.57)	
**Gilligan et al [51]**					
	Facebook	Aus $5.94 (US $4.11)		Aus $1107 (US $766.14)	
**Barney et al^b^ [84]**					
	Facebook and Instagram	US $42.21		US $21,867	
**Moseson et al^b^ [85]**					
	Facebook	US $49.48		—	
	Reddit	US $182.78		—	
**Salvy et al^b^ [20]**					
	Facebook	US $334		US $9020	
**Stuart and Moore^b^ [96]**					
	Facebook	US $1.78		US $952.81	
**Cho et al^b^ [97]**					
	Facebook	—		US $120,000	
	Facebook	—		US $215	
**Spahrkäs et al^b^ [89]**					
	Facebook	€22.42 (US $22.94)		€17,000 (US $17,397.12)	

^a^Not available.

^b^Records from the updated search.

**Table 4:** Cost of other recruitment methods.

Studies and other strategies		Cost per person	Total cost
**Ahmed et al [39]**			
	Radio	—^a^	US $12,030
**Wilkerson et al [82]**			
	Website banner advertisements	US $172.50	US $1380
**Gioia et al [52]**			
	Print newspaper	US $116.88	US $33,311
**Musiat et al [64]**			
	Recruitment agency	Aus $100 (US $69.21)	—
	Google advertisements	Aus $195.83 (US $135.53)	—
**Frandsen et al [50]**			
	Newspaper advertisements	Aus $21.52 (US $14.89)	Aus $2065.46 (US $1429.47)
**Byaruhanga et al [35]**			
	Gumtree	Aus $7.29 (US $5.05)	Aus $36.43 (US $25.21)
	Web promotions and internet searches	Aus $43.76 (US $30.29)	Aus $437.56 (US $302.83)
	Emails	Aus $128.67 (US $89.05)	Aus $2315.98 (US $1602.85)
	Newspaper	Aus $50.28 (US $34.80)	Aus $2363.38 (US $1635.65)
	Radio (interviews)	Aus $102.78 (US $71.13)	Aus $205.55 (US $142.26)
	Magazine	Aus $85.41 (US $59.11)	Aus $170.81 (US $118.21)
	Posters	Aus $566.65 (US $392.17)	Aus $566.65 (US $392.17)
	Flyers	Aus $2546.29 (US $1762.24)	Aus $2546.29 (US $1762.24)
	Telephone	Aus $3990.84 (US $2761.99)	Aus $3990.84 (US $2761.99)
**Harris et al [55]**			
	Access to organizational websites	—	Aus $5890 (US $4067.96)
	Posters	—	Aus $195 (US $134.96)
	Face-to-face events	—	Aus $43,000 (US $29,698.17)
	Conference promotion	—	Aus $44,040 (US $30,416.45)
**Moreno et al [18]**			
	In person	US $19.09	—
**Wilkerson et al [83]**			
	Mobile banner advertisements	US $375	US $3000
	Browser banner advertisements	US $187.50	US $1500
**Guthrie et al [54]**			
	Mailings	US $356 per randomized participant	US $98,682
**Waltman et al [106]**			
	Provider letter	US $29.36	US $1703
	Postcards	US $926.96	US $43,567.49
	Newspaper advertisements and television interviews	US $330.12	US $1650.63
**Watson et al [81]**			
	Press releases	—	US $1995
	Mailed letters	—	US $4054
	Google advertisements	US $34.71 (randomized)	US $3506
	Web-based survey company	—	US $7644
**Derrick et al [44]**			
	Targeted mailing	US $303 per couple	US $3635
**Carter-Harris et al [117]**			
	Newspaper advertisements	—	US $1224
**Jones et al [95]**			
	On ground	US $149.62 (randomized)	—
**Frandsen et al [48]**			
	Newspaper advertisements	Aus $52.33 (US $36.22)	Aus $4343.10 (US $3005.78)
**Juraschek et al [58]**			
	Mailed brochure	US $799	US $51,950
	Periodicals	US $437	US $10,906
**Iott et al [57]**			
	Email groups	US $10.40	US $62.37
	Personal networking	US $10.40	US $20.79
	Unified staff	US $30.32	US $727.65
	Bar outreach	US $1621.62	US $1621.62
	Flyer per palm card	US $83.20	US $416
	Publishing article in newsletter	—	US $20.79
**van Gelder et al [77]**			
	Google AdWords	€54.28 (US $55.52)	€325.66 (US $333.12)
	Care providers	—	—
**Alley et al [70]**			
	Google AdWords	Aus $495 (US $342.58)	Aus $495 (US $342.58)
	Posters	Aus $52 (US $35.99)	Aus $574 (US $397.26)
	Health care leaflets	Aus $66 (US $45.68)	Aus $990 (US $685.16)
	Letterbox drop	Aus $135 (US $93.43)	Aus $2425 (US $1678.30)
	Newspaper advertisement	Aus $145 (US $100.35)	Aus $726 (US $502.45)
	Community calendar	Aus $12 (US $8.30)	Aus $70 (US $48.45)
	Newspaper article	Aus $3 (US $2.08)	Aus $53 (US $36.68)
**Gilligan et al [51]**			
	Social networks, flyers, websites, posters, recruitment cards, email, and media coverage	Aus $58.70 (US $40.63)	Aus $4349 (US $3009.87
**Barney et al^b^ [84]**			
	Clinic-based and in person	US $865.93	US $102,180
**Moseson et al^b^ [85]**			
	Google advertisements	US $265.93	—
**Salvy et al^b^ [20]**			
	Targeted mailings	US $217	US $2387
	In-person recruitment	US $290	US $11,328
**Stuart and Moore^b^ [96]**			
	Association journal	US $375.00	—
**Cho et al^b^ [97]**			
	Personal outreach	—	—
	Public outreach	—	US $1686.04
**Spahrkäs et al^b^ [89]**			
	—	—	—

^a^Not available.

^b^Records from the updated search.

### Best Practices and Strategies Used to Enhance Social Media Recruitment

Diverse advertisement strategies are adopted by researchers when recruiting research participants through social media platforms. Each social media platform advertisement differs in specification, advertisement content, word count, and design language [47]. In the included studies, researchers identified and used one or multiple paid, targeted advertisement campaigns with different themes to reach potential participants on various platforms within a specific advertisement duration. Some models of advertisement included the use of paid targeted advertisement [18,30,31, 37,39-44, 46-52,54, 58-60,63, 65,69-75, 77,78,80, 81,91,93, 95,102,109, 113,114,117,119,120]. In addition, some researchers used untargeted advertisements [38,62,111], untargeted but paid advertisements [45,74], “boosted” posts [94,106], posts [34,61,76,90,105,110,115], both advertisements and posts [55,57,64,68], tweets [79,104,108], targeted advertisements and posts [36], advertisements [56], blasts [33], paid and unpaid social media channels [32,35], and messengers [92] to strategically advertise and recruit their potential participants.

Another identified strategy was the use of cost-related model strategies to determine the cost of the advertisements. Researchers who are engaged with any social media platform to advertise and recruit participants are billed by cost per click, cost per thousand impressions, cost per view, or cost per action or per conversion [125]. With the cost per click model, researchers are billed when a potential participant clicks on the advertisement. This approach was one of the most preferred models for researchers in the included studies used for advertising. The cost per click model budget is set at a daily, weekly, or lifetime spending limit depending on the researcher’s choice [18,30,33,38, 40,41,46, 47,48,51, 54,55,59, 60,64,69, 71,73,74, 75,77,80, 93,102, 111,126,129].

Researchers also noted considerations related to the display and design of an advertisement for a desktop application, which differed from that of a mobile app, and this affected how participants viewed and reacted to the recruitment advertisement. The displayed advertisements targeted either the user’s browser or the newsfeed [91,117]. For Facebook, the advertisement is displayed on the user’s web browser [58] or on the right-side panel of the Facebook newsfeed or placed directly in the newsfeed [47,55,65]. For Instagram, images are displayed in a linear format. Snapchat images are displayed using the story feature [47], and on Grindr, advertisements are displayed as pop-ups [57]. Therefore, cost is an influencing factor that determines the placement of the advertisement and the social media application of choice, thereby influencing the decisions that researchers make regarding recruitment.

Some researchers identified ethical challenges inherent to social media recruitment, such as privacy, confidentiality, and informed consent, and provided strategies to minimize the challenges for the researcher and the potential participant. The strategies offered and reported in the included studies included the use of a study-specific page for recruitment [2,51,76,78,82,91,94,107,112,113] and the use of secure landing sites or study webpages for data collection [35,43,48,50,58,75,106,118]. In addition to the strategies proposed by health researchers, there were 4 reports identified in the included studies that outlined guidelines and recommendations for social media recruitment (Table 5).

**Table 5:** Recommendations for best practices on social media recruitment.

Study	Study purpose or aim	Key findings	Recommendation
Curtis [118] (the United States)	To outline ethical challenges associated with social media recruitment	Social media platforms are challenged with issues of confidentiality, informed consent, and privacy issues.	Recruiting participants through secure landing sites; researchers regularly reviewing social media websites for regular updates; verifying participants’ age through cross-checking with other information may provide solutions to the challenges; setting web-based quizzes to test participant competency; and providing a summary of the research study via email
Kamp et al [122] (the United States)	To examine and describe the challenges of the Facebook recruitment method and provide recommendations	The Facebook platform presents an inherent challenge with privacy, data security, and recruiting participants.	Researchers can implement a multifactor authentication process to access research data and regularly review the privacy settings and policies on social media sites
Gelinas et al [26] (United States)	To examine the norms governing social media recruitment and analyze the ethics of recruiting, and the implication of web-based communication	The foundational norms in research ethics include respect for persons, beneficence, and justice; however, in social media, the key norms governing social media recruitment include respect for privacy and researcher’s transparency. The lack of regulatory guidance on ethics in social media recruitment poses a risk for ethical issues.	The authors proposed a checklist that researchers can use in social media recruitment
Bender et al [121] (Canada)	To develop a framework on ethics and privacy for social media and internet recruitment	The Privacy by Design framework evaluates the privacy strengths, thereby providing privacy protection in web-based recruitment.	Adhering to the Privacy by Design framework, which provides privacy-enhancing measures such as developing privacy notices, disabling comment features, or monitoring comments and removing identifiable information before it becomes public

## Discussion

### Principal Findings

Although we intentionally excluded 13 reviews from this study, 7 (54%) of them are discussed here against the findings of our review. The results from our review show an increased interest in using social media for research recruitment by researchers from different health disciplines in which social media strategies have fulfilled researchers’ recruitment needs. Considering the wide range of publications, the scope of this literature review, and the social media applications examined in this review, it can be concluded that the use of social media is on the rise, as evidenced by the increase in the number of publications in the past few years. The different research methods identified in the included studies suggest increased use of social media for a variety of research methods. Notably, a few of the included studies recruited participants for clinical studies, with most recruiting participants for cross-sectional studies. Despite social media’s reach within a broad geographic location, health researchers are still challenged with participant recruitment for clinical trials. This suggests that social media may be best suited for recruiting participants for noninterventional studies. Researchers recruiting for clinical trials may have to diversify their recruitment strategies to reach their recruitment goals until a comprehensive strategy to navigate social media platforms is established. This finding is similar to that of the review by Topolovec-Vranic and Natarajan [127], which found that only a few studies used social media to recruit participants for interventional studies as opposed to observational studies. Although the Topolovec-Vranic and Natarajan review [127] used a smaller number of studies to draw this conclusion, their findings are still significant, considering this review.

Researchers from different health disciplines, including nursing, medicine, public health, mental health, and pharmacy, have used social media for recruitment and have published their findings in a variety of journals. Some of these journals are discipline specific or interdisciplinary. This suggests different avenues for health researchers to publish their work. Within the nursing discipline, nurses are increasingly using social media for the recruitment of research studies, as published in multiple nursing and nonnursing journals. However, there are opportunities to continue promoting the use of social media among nurses for research and educational purposes.

On the basis of this review, researchers used different social media applications to advertise and recruit potential research participants. The preferred social media applications were Facebook, Instagram, Snapchat, LinkedIn, Twitter, Grindr, Reddit, Tumblr, WhatsApp, Craigslist, YouTube, and blogs to be used either solely to recruit or in conjunction with other recruitment strategies to achieve recruitment and study goals. Owing to its popularity among users and global penetration, Facebook was the most widely used application among researchers. Different social media applications enabled researchers to recruit participants with different demographics and characteristics. For instance, Facebook was used to recruit younger participants [77,113] and older individuals [67], whereas other researchers recruited young people through Tumblr [61]. This finding is similar to that of Arigo et al [128], who identified web-based platforms such as social networking sites (Facebook, Twitter, Instagram, Tumblr, and LinkedIn) as some of the common platforms that health researchers use to recruit research participants, including a diverse population for their research studies. In addition, researchers used multiple approaches and strategies to recruit participants. An approximately equal number of participants were recruited through social media alone or social media in conjunction with other strategies. This finding agrees with the general literature on the increasing acceptance of digital platforms for recruitment and with some health researchers using social media and traditional methods [129] for recruitment. In addition to the findings in this review, reviews conducted by some researchers [15,127,130-134], although focusing on only one social media application or using the term “social networking sites” broadly in their research, exclusively and comprehensively reported on a wide range of different social media applications used in research recruitment.

The different social media platforms used to target the different groups of populations such as the general population, hard-to-reach populations, and specialized populations, depended on the research aim. The hard-to-reach populations included people with addictions, sensitive health issues, ethnic groups, and poor and stigmatized populations [135]. Social media was found to be effective in reaching and recruiting hard-to-reach potential participants who were otherwise unreceptive to traditional recruitment methods because of their conditions and representations within their communities and society [50]. Researchers must weigh all available options to determine the best approach to proceed when recruiting from these populations.

The age group distribution of the research participants included in this review spanned different age categories. As shown in Table 1, the most targeted population from an age perspective was young adults. According to Kemp [10], the engagement of social media platforms among youth stands at 58% between the ages of 16 and 24 years. This is not surprising because of the acceptability of social media among youth who are considered technology savvy and their tendency to use social media regularly. As such, targeting such an age group for research studies can lead to increased participation. An observation of interest in this review is the low involvement of children and adolescents aged <18 years and older adults. Only 2% (2/96) of studies [18,120] involved adolescents between the ages of 13 and 14 years. As researchers require parental consent among the children and the adolescent group, research studies involving these groups are relatively limited. This finding is similar to that of Amon et al [130], who suggested that instead of focusing on adolescents who require parental consent, targeting parents or guardians of the intended group could help waive parental consent.

For older adults, the usability of social media platforms presents a challenge, such as platform design and content, as these platforms are tailored to the interests of the younger population [136]. Owing to the complex design, nature, and privacy-related concerns associated with social media platforms, older adults are more comfortable and familiar with traditional forms of recruitment than social-mediated platforms [137]. Other barriers encountered by older adults include intrapersonal, interpersonal, functional, and structural elements that hinder the use of social media platforms [138]. Although social media presents a challenge for recruitment in the older population, researchers can continue to explore traditional methods in such populations to offer an equal chance of participation in research studies. The trade-off between using traditional methods and social media for recruiting research participants is a complicated issue, requiring health researchers to weigh options and the benefits and risks to the participant and the research study, as well as more creative ways of engaging low participating groups.

There is a debate on the cost-effectiveness of social media in the literature. Some studies found the social media method to be cost-effective, whereas other studies disagree with this assertion [18,58]. In this review, the cost of other recruitment strategies compared with social media recruitment strategies was presented as part of this review to assist researchers in making an informed decision (Tables 3 and 4). In addition, in this review, the factors that influenced the cost associated with recruitment varied from one study to the next. Some researchers reported advertising, recruitment, and other administrative costs as the total cost, whereas others reported only aspects of social media advertisement and recruitment as the total cost. Owing to the inconsistency in cost reporting, having a standardized cost reporting system to maintain consistency would help to effectively determine whether social media recruitment is cost-effective. On the basis of the analysis of the cost-effectiveness of both social media and other recruitment strategies, this review found that social media was viewed by researchers as a cost-effective strategy. Although 28% (27/96) of studies in this review reported on the cost of social media compared with other recruitment methods, not all researchers found social media as a cost-effective method. Nonetheless, given that a large proportion of these studies found social media to be cost-effective, this review supports this conclusion. Compared with the previously published reviews by Reagan et al [15] and Topolovec-Vranic and Natarajan [127], this review provides additional insights and includes a broader range of studies. This review captured additional literature not included in the review by Reagan et al [15], which relied only on 18 articles, of which only 10 articles reported on cost. In the review by Topolovec-Vranic and Natarajan [127], the authors included 30 studies, of which 5 reported on cost-effectiveness, and 7 did not find social media to be a cost-effective method. The findings also revealed that the cost of recruitment for hard-to-reach populations differs from that for the general population. Jurascheck et al [58] found that recruiting through Facebook advertisements for the African American population was costly; however, advertisements were effective in directing eligible participants to the website. Hence, researchers hoping to recruit research participants through social media must consider these factors to make decisive choices on the most suitable method for recruitment.

### Best Practices for the Use of Social Media in Recruitment of Research Participants

Researchers are increasingly tapping into the available opportunities to use social media platforms for their research studies. However, there is a need for best practices to guide this process. To adequately explore and navigate social media platforms successfully for recruitment, adhering to best practices, including those of ethical considerations (informed consent, privacy, confidentiality, and transparency) that protect the researcher and participants, is of utmost importance [26,118,122]. In the review by Amon et al [130], the authors found that participants recruited on web-based platforms were subjected to the same ethical standards as though they were responding to a traditional recruitment method. In that regard, Gelinas et al [26] were of the view that REBs should standardize social media techniques by clarifying their similarity to traditional recruitment. Furthermore, the findings from the review also establish the need to take additional steps to make available informed consent through other means, where the potential participants are well informed with detailed information about the research study before participation. To curb and curtail the complexities and complicated nature of informed consent, the findings from this review support the recommendations suggested by Herbell and Zauszniewski [94] and Stokes et al [105] in their studies to make an information sheet in a downloadable version available for participants and send web-based consent forms to potential participants after meeting the eligibility criteria. To maintain the confidentiality of both researchers and participants, Shaver et al [71] suggested using anonymous surveys and directing interested participants through a survey link to a landing page for study information. Researchers are discouraged from directly recruiting participants on social media platforms but instead using the social media platform to advertise, as the confidentiality and privacy of participants’ data cannot be guaranteed. To further ensure the provision of privacy, Bender et al [121] used privacy-enhancing measures aligned with the principles of Privacy by Design by disabling the comment feature, developing privacy notices for social media campaigns, sending disclaimers about the privacy risks of social media pages, and building privacy protection into the recruitment strategy. Although the tenets of the foundational principles were incorporated to avoid privacy-related issues, Bender et al [121] were of the view that the principles of transparency and user-centric options of Privacy by Design provide inadequate guidance on how to design privacy notices using these key principles.

In addition, some factors were identified to influence advertisements, such as advertisement targets, crafting of multiple advertisement campaigns with different wordings and themes, rotating and alternating advertisements, payment model, duration of the advertisement, and location of the advertisement on the social media platform. To favorably achieve the results of recruiting an increasing number of participants for research, researchers advertising on social media must strategically reach out to their participants. On the basis of the findings of this review, using an appealing image and simple and consistent language through both the text caption and image [69] influences and attracts participants to the study. Some social media platforms’ advertising policies provide details on advertisement content, including the choice of words and counts and the duration of an advertisement on their platforms. The advertisement policies differ from platform to platform. Researchers must research any platform they wish to engage in, understand the policies, and adhere to them. In addition, working with REBs on social recruitment messages and strategies helps avoid ineffective strategies and enhance ethical conduct. Incorporating prescreening questions before allowing participants to enter details for study participation reduces the rate of ineligible participants and maximizes the reach and sample representativeness. Researchers can use these applications simultaneously because of the feasibility of incorporating social media platforms such as Instagram, Snapchat, and Facebook into a study without difficulty [47].

### Implications

The findings from the review show the increasing accessibility and multifunctionality of social media platforms that could be leveraged to further support health science research. In fact, one of the benefits of social media for conducting research recruitment has been amplified during the COVID-19 pandemic because of the limitations to in-person recruitment, thus sustaining the continuity of research.

Generally, social media platforms provide avenues for a practical approach to reaching diverse, extensive, and targeted audiences [139] or populations, particularly those that are hard to reach. Further research may be needed to understand the barriers to and facilitators of older adults’ engagement with social media platform recruitment.

Although different approaches to recruitment, advertisement, cost determination, and efficiency reporting can challenge novice researchers planning to use social media, there are ways of mitigating some of these challenges. For example, with the availability of funds and resources, researchers can benefit from hiring specialized companies or third-party service organizations to assist with the marketing and development of social media recruitment strategies and other innovative recruitment approaches targeting potential research participants. It is also recommended that these strategies be discussed and coordinated with the researcher’s academic institution’s REB to ensure no risks to participants.

The lack of explicit regulations by REBs to guide researchers continues to prevent the full exploration of social media platforms to support health science research. As such, stakeholders and collaborative efforts from research-based organizations, academia, researchers, think tanks, and student groups must partner to develop guidelines that reflect the use of social media in research studies. The different guidelines developed and published by researchers and academic institutions can provide a context for what is available. Therefore, based on our review, we propose a tentative description or guideline to guide researchers based on what we have synthesized from the literature included in this review (Multimedia Appendix 6). Ultimately, this guide could serve as a starting point to inform stakeholders in the development of a standardized protocol to guide health science researchers in the use of various social media platforms for research participant recruitment.

Finally, there are opportunities to advance health science education regarding social media use in general and its use for the recruitment of research participants. As students become technologically savvy, incorporating social media into their learning process will allow them to effectively engage with the platform. Schools can also provide guidelines on social media platforms on their websites to enhance learning about their applications in research processes. In addition, teaching students about best practices that support professional social media use and including social media applications as part of ethics training programs are also recommended.

### Strengths and Limitations

The findings of this review offer a broad perspective on the use of social media platforms for participant recruitment by health researchers. A large number of studies were included for analysis in this review. The timelines for the included studies span >20 years and provide sufficient time to capture all studies published during the popularity of social media. This study comprehensively synthesized available literature from all health science disciplines. However, the review was limited to studies reported only in English.

### Conclusions

The purpose of this integrative review was to explore the literature on recruiting participants for research studies through social media application tools and identify best practices to assist researchers in conducting research participant recruitment via social media tools. This integrative review expanded on the review by Reagan et al [15], which focused primarily on Facebook, by including other social media applications used by health researchers to recruit research participants, such as Facebook, Craigslist, Instagram, LinkedIn, Reddit, Tumblr, Twitter, and YouTube. Overall, the findings showed that social media is a suitable, viable, and cost-effective channel for recruiting research participants, despite some challenges associated with its use. Health researchers are increasingly embracing various social media platforms in their research to recruit participants from various age groups and diverse backgrounds; however, there is less use of social media to recruit older adults. Adhering to best practices when targeting various populations through social media advertisements is vitally important to protect participants’ and researchers’ rights and increase participation. REBs must proactively provide protocols and best practice guidelines that researchers can apply during the advertisement and recruitment of research participants.

## Appendix

Multimedia Appendix 1 Top 10 Universities in Canada QS World University Rankings 2021.

Multimedia Appendix 2 Search strategy for keywords and subject headings of the search terms used in each database.

Multimedia Appendix 3 Characteristics of included studies.

Multimedia Appendix 4 Critical Appraisal Skills Program critical appraisal tools used to critically appraise and evaluate each of the included studies.

Multimedia Appendix 5 List of excluded studies.

Multimedia Appendix 6 Tentative guidelines to assist health sciences researchers in social media recruitment.

## Abbreviations

PRISMA: Preferred Reporting Items for Systematic Reviews and Meta-Analyses
REB: research ethics board
