# Supplementary material for: Social Media Use for Research Participant Recruitment: Integrative Literature Review
Source: J Med Internet Res. 2022 Aug 4;24(8):e38015. doi: 10.2196/38015 (PMC9389385; doi:10.2196/38015)
Supplement: Multimedia Appendix 1 [file jmir_v24i8e38015_app1.docx]

Multimedia Appendix 1: Top 10 Universities in Canada -QS World University Rankings 2021

| Canada Rank | Global Rank | University | City | Province | Name of Research Ethics Board | Guidance on Social Media Recruitment | Link to the website |
| --- | --- | --- | --- | --- | --- | --- | --- |
| 1 | 25 | University of Toronto | Toronto | Ontario | Health Sciences Research Ethics Board | Not available | Not available |
| 2 | 31 | McGill University | Montreal | Quebec | McGill University Health Centre for Applied Ethics | Not available | Not available |
| 3 | 45 | University of British Columbia | Vancouver | British Columbia | University of British Columbia Clinical Research Ethics Board | Social networking sites (Draft) | See the footnote below |
| 4 | 118 | University of Montreal (Université de Montréal) | Montreal | Quebec | Research Ethics, Ethics and Population Health, Ethics and Global Health | Not available | Not available |
| 5 | 119 | University of Alberta | Edmonton | Alberta | University of Alberta Research Health Ethics Board | 1. Social media/online recruitment.  2. Internet research  3. Email for recruitment | See the footnote below |

Multimedia Appendix 1: *continued*

| Canada Rank | Global Rank | University | City | Province | Name of Research Ethics Board | Guidance on Social Media Recruitment | Link to the Website |
| --- | --- | --- | --- | --- | --- | --- | --- |
| 6 | 144 | McMaster University | Hamilton | Ontario | Hamilton Integrated Research Ethics Board (HiREB) | Not available | Not available |
| 7 | 166 | University of Waterloo | Waterloo | Ontario | Research Ethics Committee | Not available | Not available |
| 8 | 203 | Western University | London | Ontario | The Health Sciences Research Ethics Board (HSREB) | 1.Social Media  2. Email  3. Survey panel  3. SONA Recruitment Database | See the footnote below |
| 9 | 246 | University of Calgary | Calgary | Alberta | Conjoint Health Research Ethics Board (CHREB) | Not available | Not available |
| 9 | 246 | Queen’s University | Kingston | Ontario | The Queen's University Health Sciences and Affiliated Teaching Hospitals Research Ethics Board (HSREB) | Not available | Not available |

*Notes.*

University of British Columbia Link to Website for the Guideline:

ethics.research.ubc.ca/sites/ore.ubc.ca/files/documents/social_networking_sites-GN-June_2012.pdf

University of Alberta Link to Website for the Guideline:

<https://www.ualberta.ca/research/research-support/research-ethics-office/human-research-ethics/recruitment/online.html>

Western University Link to Website for the Guideline:

<https://www.uwo.ca/research/_docs/ethics/hsreb_guidelines/Guidelines_for_Participant_Recruitment_vSept242018.pdf>
