# Supplementary material for: Social Media Use for Research Participant Recruitment: Integrative Literature Review
Source: J Med Internet Res. 2022 Aug 4;24(8):e38015. doi: 10.2196/38015 (PMC9389385; doi:10.2196/38015)
Supplement: Multimedia Appendix 2 [file jmir_v24i8e38015_app2.docx]

Multimedia Appendix 2

Overview of Search Terms

| Database | Search ID# | Search Terms | No. of Record Retrieved on Oct 11, 2020 | No. of Records Retrieved on Feb 24, 2022 |
| --- | --- | --- | --- | --- |
| CINAHL Plus with Full Text | S1 | Digital* or Virtual* or Virtual world*or Online or Email or Facebook or Facebook Messenger or Instant Messaging or Social Media or Social Web or Instagram or Twitter or tweet* or Snapchat or Craigslist or Online System* or Online forum*or Pinterest or Tumblr or User Generated Content or Internet or Online Social Network* or Social Network* or LinkedIn or Tumblr or Reddit or WhatsApp or Website* or Web site* or Web2 or Web 2.0 or Blog* or Microblog* or Webcast* or Web log or Video Sharing or Myspace or Podcast* or Pod-cast*or Patient support group* or Online communit* | 230,767 | 275,286 |
|  | S2 | TI ((participant* or subject* or research* or Advertis* or Advertiz*) N3 recruit*) | 541 | 596 |
|  | S3 | (MM “Research Subject Recruitment”) | 2,621 | 2,876 |
|  | S4 | S2 OR S3 | 2886 | 3,183 |
|  | S5 | S1 AND S4 | 393 | 478 |
|  | S6 | S1 AND S4  Limiters - Scholarly (Peer Reviewed) Journals  Published Date: 20000101 – 20201031 | 358 | 69 |
|  | S7 | S1 AND S4  Limiters – Scholarly (Peer Reviewed); Journals Published Date: 20000101 – 20201031; English Language | 355 | N/A |
|  | S8 | Limiters – Scholarly (Peer Reviewed); Journals Published Date: 20201101 – 20220224; English Language | NA | 65 |
|  |  | Total 420 | | |

Multimedia Appendix 2. (*continued)*

| Database | Search ID# | Search Terms | No. of Record Retrieved on Oct 11, 2020 | No. of Records Retrieved on Feb 24, 2022 |
| --- | --- | --- | --- | --- |
| Medline (Ovid) | 1 | (Digital* or Virtual* or Virtual world*or Online or Email or Facebook or Facebook Messenger or Instant Messaging or Social Media or Social Web or Instagram or Twitter or tweet* or Snapchat or Craigslist or Online System* or Online forum*or Pinterest or Tumblr or User Generated Content or Internet or Online Social Network* or Social Network* or LinkedIn or Tumblr or Reddit or WhatsApp or Website* or Web site* or Web2 or Blog* or Microblog* or Webcast* or Web log or Video Sharing or Myspace or Podcast* or Pod-cast*or Patient support group* or Online communit*) .mp. [mp=title, abstract, original title, name of substance word, subject heading word, floating sub-heading word, keyword heading word, organism supplementary concept word, protocol supplementary concept word, rare disease supplementary concept word, unique identifier, synonyms] | 476853 | 523135 |
|  | 2 | ((participant* or subject* or research or advertis* or advertiz*) adj3 recruit*).ti | 642 | 664 |
|  | 3 | Research Subject Recruitment.mp. | 58 | 61 |
|  | 4 | 2 or 3 | 681 | 706 |
|  | 5 | 1 and 4 | 167 | 174 |
|  | 6 | Limit 5 to (English language and yr= “2000 – 2020”) | 164 | N/A |
|  | 7 | Limit 5 to (English language and yr= “2021 – 2022”) | N/A | 5 |
|  |  | Total | 169 | |

Multimedia Appendix 2. *(continued)*

| Database | Search ID# | Search Terms | No. of Record Retrieved on Oct 11, 2020 | No. of Records Retrieved on Feb 24, 2022 |
| --- | --- | --- | --- | --- |
| PSYCInfo | 1 | Digital* or Virtual* or Virtual world*or Online or Email or Facebook or Facebook Messenger or Instant Messaging or Social Media or Social Web or Instagram or Twitter or tweet* or Snapchat or Craigslist or Online System* or Online forum*or Pinterest or Tumblr or User Generated Content or Internet or Online Social Network* or Social Network* or LinkedIn or Tumblr or Reddit or WhatsApp or Website* or Web site* or Web2 or Blog* or Microblog* or Webcast* or Web log or Video Sharing or Myspace or Podcast* or Pod-cast*or Patient support group* or Online communit*.mp. [mp=title, abstract, heading word, table of contents, key concepts, original title, tests & measures, mesh] | 166413 | 187998 |
|  | 2 | ((participant* or subject* or research or advertis* or advertiz*) adj3 recruit*).ti | 265 | 282 |
|  | 3 | Research Subject Recruitment.mp. | 26 | 28 |
|  | 4 | 2 or 3 | 284 | 302 |
|  | 5 | 1 and 4 | 56 | 63 |
|  | 6 | limit 5 to (english language and yr= “2000 -2020”) | 54 | N/A |
|  | 7 | limit 5 to (english language and yr= “2021 -2022”) | N/A | 0 |
|  |  | Total | 54 | |

Multimedia Appendix 2. *(continued)*

| Database | Search ID# | Search Terms | No. of Record Retrieved on Oct 11, 2020 | No. of Records Retrieved on Feb 24, 2022 |
| --- | --- | --- | --- | --- |
| EMBASE (Ovid) | 1 | Digital* or Virtual* or Virtual world*or Online or Email or Facebook or Facebook Messenger or Instant Messaging or Social Media or Social Web or Instagram or Twitter or tweet* or Snapchat or Craigslist or Online System* or Online forum*or Pinterest or Tumblr or User Generated Content or Internet or Online Social Network* or Social Network* or LinkedIn or Tumblr or Reddit or WhatsApp or Website* or Web site* or Web2 or Blog* or Microblog* or Webcast* or Web log or Video Sharing or Myspace or Podcast* or Pod-cast*or Patient support group* or Online communit*.mp. [mp=title, abstract, heading word, drug trade name, original title, device manufacturer, drug manufacturer, device trade name, keyword, floating subheading word, candidate term word] | 622789 | 720454 |
|  | 2 | Research Subject Recruitment.mp. | 70 | 79 |
|  | 3 | ((participant* or subject* or research or advertis* or advertiz*) adj3 recruit*).ti | 755 | 832 |
|  | 4 | 2 or 3 | 809 | 893 |
|  | 5 | 1 and 4 | 193 | 227 |
|  | 6 | Limit 5 to (English language and yr=”2000 -2020”) | 190 | N/A |
|  | 7 | Limit 5 to (English language and yr=”2021 -2022”) | N/A | 19 |
|  |  | Total | 209 | |

Multimedia Appendix 2. *(continued)*

| Database | Search ID# | Search Terms | No. of Record Retrieved on Oct 11, 2020 | No. of Records Retrieved on Feb 24, 2022 |
| --- | --- | --- | --- | --- |
| MEDLINE (EBSCOhost) | S1 | Digital* or Virtual* or Virtual world*or Online or Email or Facebook or Facebook Messenger or Instant Messaging or Social Media or Social Web or Instagram or Twitter or tweet* or Snapchat or Craigslist or Online System* or Online forum*or Pinterest or Tumblr or User Generated Content or Internet or Online Social Network* or Social Network* or LinkedIn or Tumblr or Reddit or WhatsApp or Website* or Web site* or Web2 or Web 2.0 or Blog* or Microblog* or Webcast* or Web log or Video Sharing or Myspace or Podcast* or Pod-cast*or Patient support group* or Online communit* | 575,805 | The database is no longer available on the University of Alberta library database. |
|  | S2 | TI ((participant* or subject* or research* or Advertis* or Advertiz*) N3 recruit*) | 756 |  |
|  | S3 | (MM “Research Subject Recruitment”) | 43,817 |  |
|  | S4 | S2 OR S3 | 11,026 |  |
|  | S5 | S1 AND S4 | 448 |  |
|  | S6 | S1 AND S4  Limiters - Date of Publication: 20000101 – 20201031 | 401 |  |
|  | S7 | SI AND S4  Limiters – Date of Publication: 20000101 – 20201031;  English Language | 369 |  |
|  |  | Total | 369 |  |

Multimedia Appendix 2. (*continued)*

| Database | Search ID# | Search Terms | No. of Record |
| --- | --- | --- | --- |
| Scopus (Advanced Search)(Oct 11, 2020) |  | *TITLE-ABS KEY (*"digital*"*OR*"virtual*"*OR*"Virtualworld*"*OR*"online"*OR*"email"*OR*"Facebook"*OR*"Facebook Messenger"*OR*"Instant Messaging"*OR*"Social Media"*OR*"Social Web"*OR*"Instagram"*OR*"twitter"*OR*"tweet*"*OR*"snapchat"*OR* "Craigslist"*OR*"Online System*"*OR*"Online forum*"*OR*"pinterest"*OR*"tumblr"*OR*"User Generated Content"*OR*"internet"*OR*"Online Social Network*"*OR*"Social Network*"*OR*"LinkedIn"*OR*"reddit"*OR*"whatsapp"*OR*"website*"*OR*"Web site*"*OR*"web 2"*OR*"Web 2.0"*OR*"blog*"*OR*"microblog*"*OR*"webcast*"*OR*"Web log"*OR*"Video Sharing"*OR*"myspace"*OR*"podcast*"*OR*"pod-cast*"*OR*"Patient support group*"*OR*"online"*OR*"communit*"*)  AND  (*"participant*"*OR*"subject*"*OR*"research"*OR*" advertiz*"*OR*"advertis*"*)*"recruit*"*W/3*"Research Subject Recruitment"*AND  ( LIMIT- 2000-*2020*)* *-TO ( LANGUAGE ,*"English"*) )* | 65 |
| (Feb 24, 2022) |  | *TITLE-ABS-KEY ( ( "digital*" OR "virtual*" OR "Virtualworld*" OR "online" OR "email" OR "Facebook" OR "Facebook Messenger" OR "Instant Messaging" OR "Social Media" OR "Social Web" OR "Instagram" OR "twitter" OR "tweet*" OR "snapchat" OR "Craigslist" OR "Online System*" OR "Online forum*" OR "pinterest" OR "tumblr" OR "User Generated Content" OR "internet" OR "Online Social Network*" OR "Social Network*" OR "LinkedIn" OR "reddit" OR "whatsapp" OR "website*" OR "Web site*" OR "web 2" OR "Web 2.0" OR "blog*" OR "microblog*" OR "webcast*" OR "Web log" OR "Video Sharing" OR "myspace" OR "podcast*" OR "pod-cast*" OR "Patient support group*" OR "online" OR "communit*" ) AND ( "participant*" OR "subject*" OR "research" OR " advertiz*" OR "advertis*" ) AND "recruit*" W/3 "Research Subject Recruitment" ) AND PUBYEAR > 2021 AND PUBYEAR < 2022 AND ( LIMIT-TO ( LANGUAGE , "English" ) )* | 0 |
|  |  | Total | 65 |

*Notes.* The *symbol refers to truncation of a search term

Legends: Grey areas show the change in Scopus syntax

Abbreviations: N/A, not applicable
