# Supplementary material for: Social Media Use for Research Participant Recruitment: Integrative Literature Review
Source: J Med Internet Res. 2022 Aug 4;24(8):e38015. doi: 10.2196/38015 (PMC9389385; doi:10.2196/38015)
Supplement: Multimedia Appendix 3 [file jmir_v24i8e38015_app3.docx]

Multimedia Appendix 3. Characteristics of Included Studies

| Author  (Country) | Study design | Population &  Total n | Total n (%) enrolled/  Recruited through SM | SM platform | Other  recruitment strategies | Ads paid/not paid | Incentive/ funded | Limitations of SM | Duration of ads |
| --- | --- | --- | --- | --- | --- | --- | --- | --- | --- |
| Ahmed et al. 2020 [39] (USA) | Cross-sectional study | Patients with ASD & their families; n= 374 | 139 (37.2) | Facebook | Radio | Yes | NR; F | NR | SM– 8 WK; OM- 4 WK |
| Cowie & Gurney. 2018 [43] (USA) | Clinical trial | Healthy aged ≥ 60 years; n= 857 (inquires) | 45 (5.2) | Facebook | Website and intranet, referral, print, flyers, billboards, direct mailings, newspapers, word-of-mouth, recruitment events, posters | Paid | NR; F | NR | SM- 8 WK; OM- 11 WK |
| Chambers et al. 2020 [109] (USA) | Qualitative study | Emergency department and intensive care registered nurses; ≥ 21 years; n=17 | 0 | Facebook | Publishing agency, snowing strategy | Paid | I; NR | NR | SM- 14 D; OM-15 & 31 D |
| Thornton et al. 2016 [74] (Australia) | Cross-sectional study | Australians ≥ 18 years with alcohol, cannabis, and drug addictions; n= 942 | 524 (56.0) | Facebook | Research database, University course work | Paid | I; F | NR | SM- 26 D;  OS- NR |
| Nash et al. 2017 [65] (Australia) | Secondary analysis | 18-69 years; n= 67 | 67 (100) | Facebook | Radio, newspaper, poster | Paid | I; F | NR | SM- 4 M.  OM- NR |
| Gioia et al. 2016 [52] (USA) | Secondary analysis | Alcohol consumers ≥ 21 years; n=473 | 188 (40.0) | Craigslist | Print newspaper | Paid | I; F | NR | SM- 2 WK |
| van Gelder et al. 2019 [77] (The Netherlands) | Cohort study | Pregnant women 18-45 years; n=392 | 59 (15.0) | Facebook | Google AdWords, Prenatal care providers | Paid | NR; F | NR | SM- 27-31 D; OS- 30 D |

Multimedia Appendix 3. *(continued)*

| Author (Country) | Study design | Population  & Total n | Total n (%) enrolled  /Recruited through SM | SM  platform | Other  recruitment  Strategies | Ads paid/  Not paid | Incentive/ funded | Limitations of SM | Duration of ads |  |
| --- | --- | --- | --- | --- | --- | --- | --- | --- | --- | --- |
| Close et al. 2013 [42] (USA) | Cross-sectional study | Boys 8 and 18 years with Klinefelter syndrome; n= 43 | 16 (37.2) | Facebook | Recruit Source, patient advocacy group, support groups, teleconference, traditional recruitment. | Paid | NR; NR | NR | SM- 7D; OM- NR |  |
| Burrell et al. 2012 [33] (USA) | Clinical trial | MSM ≥ 18 years; n=105 | 24 (23%) | Grindr | Traditional means | NR | NR; F | NR | NR |  |
| Corey et al. 2018 [112 (USA) | Secondary analysis | Former family caregivers; ≥ 18 years; n=171 | 53 (31.0) | Facebook | Websites, referrals from professional groups | NR | NR; F |  |  |  |
| Derrick et al. 2017 [44] (USA) | Secondary analysis | Single-smoker couples; 18-55 years; n= 62 completed orientations | 45 (73.0) | Facebook | Targeted direct mailing | Paid | I; F | NR | SM- 8 & 14 M |  |
| Juraschek et al. 2018 [58] (USA) | Randomized controlled trial | Cancer survivor ≥ 18 years; n=121 | 7 (6.0) | Facebook | Directly targeted mails, referrals, word-of-mouth, community fairs, periodicals | Paid | I; F | NR | SM- 91 D |  |
| Gilligan et al. 2014 [51] (Australia) | Quasi-experimental study | Parents (≥30 years) of 13–17-year-old children; n=278 | 204  (73.4) | Facebook | Flyers, websites, posters, recruitment cards, email, media coverage, social networks | Paid | I; F | NR | SM- 4 WK; OS- NR |  |
| Hansen et al. 2016 [114] (USA) | Qualitative study | Caregivers ≥ 18 years; n=9 | 9 (100) | Social media (Facebook, illness blogs) | Community flyers | Paid | NR; F | NR | SM- 3 D, 2-4 WK |  |

Multimedia Appendix 3. (con*tinued)*

| Author (Country) | Study design | Population  &  Total n | Total n (%) enrolled/  Recruited through SM | SM platform | Other recruitment strategies | Ads paid/not paid | Incentive/ funded | Limitations of SM | Duration of ads |
| --- | --- | --- | --- | --- | --- | --- | --- | --- | --- |
| Desroches. 2020 [1130] (USA) | Cross-sectional study | Practicing nurses; 18-65 years; n=248 participants recruited. | 118 (46.0) | Facebook | Email | Paid | I; F | There is sample bias in terms of geographic diversity. The identity of participants cannot be verified. The generalization of the study is limited by the self-selection into the study | SM- 3 WK |
| Frandsen et al. 2014 [50] (Australia) | Randomized controlled trial | Tobacco smokers ≥ 18 years; n= 266 participants recruited | 138 (52.0) | Facebook | Flyers, newspaper ads, word-of-mouth | Paid | NR; F | Study participants' identity was not guaranteed. Unable to accurately compare the costs associated with advertising. | NR |
| Frandsen et al. 2016 [48] (Australia) | Secondary analysis | Smokers ≥ 18 years; n= 104 completed the survey | 50  (48.1) | Facebook | Newspaper ads, flyers, radio, interviews, word-of-mouth | Paid | NR; NR | Results cannot be generalized to other social media platforms due to the different recruitment approaches by the different platforms. | NR |
| Wilkerson et al. 2016 [82] (India) | Cross-sectional study | MSM-H ≥ 18 years; n=449 | 0 | Social media (Facebook) | Palm cards, emails, website, film festival, pass recruitment | Paid | I; F | Internet access by a certain class of persons limited the generalization of study findings | NR |
| Alley et al. 2016 [70] (Australia) | Secondary analysis | Adults ≥ 18 years engaged in physical activity; n=140 | 74 (53.5) | Facebook | Print-based, websites, Google AdWords, community calendars | Yes | I; NR | Allocated funds for ads were directed to the more successful method, Facebook. | SM – 12 M |

Multimedia Appendix 3. *(continued)*

| Author (Country) | Study design | Population &  Total  n | Total n (%) enrolled  /Recruited through SM | SM platform | Other recruitment strategies | Ads paid/not paid | Incentive/ funded | Limitations of SM | Duration of ads |
| --- | --- | --- | --- | --- | --- | --- | --- | --- | --- |
| Kelleher et al. 2018 [61] (USA) | Randomized controlled trial | Young people with depression 15-23 years; n=25 | 25 (100) | Tumblr | NA | NR | NR; F | The anonymity of the survey prevented follow-ups. | NR |
| Burgess et al. 2017 [31] (Australia) | Randomized controlled trial | Women ≥ 18 years with at last a child aged 5-12 months; n=498 | 498 (100) | Facebook, Instagram | NA | Paid | I; NR | Relying on the participants' profile information on Facebook/Instagram may not be accurate or up-to-date. | 30 D |
| Grov et al. 2013 [90] (USA) | Web-based survey | MSM ≥ 18 years; n=81 | 81(100) | Craigslist | NA | NR | I; F | Data localized to one State; therefore, findings cannot be generalized | NR |
| Dunn & Woo. 2018 [45] (USA) | Cross-sectional study | Chinese-speaking adults aged 45 years or older; n=508 | 508 (100) | Facebook, YouTube | NA | Paid | NR; NR | Limited Facebook demographic data and inaccurate participant profile information | 48 HRS |
| Cavallo et al. 2020 [38] (USA) | Cross-sectional study | Cigarillo smokers; 14-28 and 13-34 years; n=1089 | 1089 (100) | Facebook, Twitter, Instagram | NA | Paid | I; F | Twitter, Facebook, and Instagram had differences in the demographic profiles of users. | 39 WK |
| Herbell & Zauszniewski. 2018 [94] (USA) | Secondary analysis | Caregivers ≥ 18 years; n=230 | 230 (100) | Twitter, Facebook | NA | Paid | I; F | Participants’ identities cannot be verified | 2 M |
| Fenner et al. 2012 [46] (Australia) | Cross-sectional study | 16 - 25-year-old females; n=278 | 278 (100) | Facebook | NA | Paid | I; F | Low participation rate and biased participation, responses, and sampling. | NR |

Multimedia Appendix 3*. (continued)*

| Author (Country) | Study design | Population & Total n | Total n (%) enrolled/  Recruited through SM | SM platform | Other recruitment  strategies | Ads paid/not paid | Incentive/ funded | Limitations of SM | Duration of ads |
| --- | --- | --- | --- | --- | --- | --- | --- | --- | --- |
| Waltman et al. 2019 [106] (USA) | Randomized controlled trial | Postmenopausal women ≥ 19 years; n=276 | 44 (16.0) | Facebook | Community events, newspaper/television ads, healthcare provider’s letters, postcard mailings, digital ads. | Paid | NR; F | NR | NR |
| Harris et al. 2015 [55] (Australia) | Cohort study | Australian women, aged 18-23 years; n= 3,509 | NR | Facebook, Twitter | Gumtree, emails, events, posters, referrals, consumer research organizations, conference promotions | Paid | I; F | NR | SM- 8 M |
| Hsiao et al. 2020 [56] (Taiwan) | Cross-sectional study | PLHIV; n=250 enrolled | 125  (50.0) | Facebook | Hospital | NR | NR; F | NR | NR |
| Vial et al. 2014 [78] (USA) | Cross-sectional study | Adult ≥ 18 years MSM; n=2843 | 355  (12.5) | Facebook | Websites, bars, clubs, community venues | NR | NR; F | NR | NR |
| Ahmed et al. 2013 49] (Australia) | Cross-sectional study | Women, aged 16-25 years; n=278 | 278 (100) | Facebook | NA | NR | NR; F | NR | NR |
| Musiat et al. 2016 [64] (Australia) | Secondary analysis | Individuals 18-25 years; n=264 | 102 (39.0) | Twitter, Facebook, YouTube | Google Ads, recruitment agency, websites, emails, university site | Paid | NR; F | NR | SM- 10 D, 2 W, 3 M |
| Guthrie et al. 2019 [54] (USA) | Randomized controlled trial | Women aged 45-70 years; n=302 | 25 (8.3) | Facebook | Mailing | Paid | I; F | NR | SM- 15 & 28 D |
| Jones et al. 2017 [95] (USA) | Secondary analysis | African American or Black women, 18 - 29 years; n=1435 | 940 (65.5) | Facebook | On-the-ground | Paid | NR; F | NR | NR |

Multimedia Appendix 3*. (continued)*

| Author (Country) | Study design | Population & Total n | Total n (%) enrolled/  Recruited through SM | SM platform | Other recruitment strategies | Ads paid/not paid | Incentive/ funded | Limitations of SM | Duration of ads |
| --- | --- | --- | --- | --- | --- | --- | --- | --- | --- |
| Watson et al. 2018 [81] (USA) | Randomized controlled trial | Adult smokers ≥ 18 years; n= 2,637 | 1303 (49.4%) | Facebook | Google, web-based survey panel, traditional methods (press releases, newspaper, radio, and television interviews) | Paid | I; F | Limits with the generalization of findings due to the evolving nature of a technology-based advertisement. | SM- 12 M; OS- NR |
| Byaruhanga et al. 2019 [35] (Australia) | Cross-sectional survey | Tobacco smokers ≥ 18 years; n=655 | 548  (84.0%) | Facebook, Twitter, | Emails, Gumtree, website, internet, newspaper, posters, flyers, radio, magazine, telephone calls | Paid | NR; F | Internet access as an eligibility criterion may have excluded some interested participants.  Social media recruitment strategies limited the reach of participants to a small proportion of smokers from remote areas and none from very remote areas. | SM- 17 M;  OS- NR |
| Blumenberg et al. 2019 [92] (Brazil) | Cross-sectional survey | Birth cohort members; n=642 | 443  (69.0) | Facebook, WhatsApp messenger | Emails | NR | NR; F | The likelihood of eligible participants not reading their  WhatsApp messages | NR |
| Moreno et al. 2017 [18] (USA) | Randomized controlled trial | High school adolescents 14 - 18 years; n=45 | 8 (18.0) | Social media (Facebook, Twitter, Blog post) | In-person (clinics, schools) | Paid | NR; F | IRB's non-incentive promotions directive on social media complicated the study process | SM- 1 WK;  OS- NR |

Multimedia Appendix 3*. (continued)*

| Author (Country) | Study design | Population &  Total n | Total n (%) enrolled/Recruited through SM | SM platform | Other recruitment strategies | Ads paid/not paid | Incentive/ funded | Limitations of SM | Duration  of ads |
| --- | --- | --- | --- | --- | --- | --- | --- | --- | --- |
| Williamson et al. 2018 [107] (UK) | Cross-sectional study | Former and current pregnant asthmatic women; n=402 engagements | 8 | Facebook, Twitter | NA | NR | NR; NR | NR | 76 D (Twitter) |
| Cahill et al. 2019 [36] (USA) | Cross-sectional study | Users of genetic testing services. n=764 | 764 | Facebook, Twitter, Reddit | NA | Paid | NR; NR | NR | 14 D (Facebook),10 D (Twitter) |
| Burton-Chase et al. 2017 [34] (USA) | Cross-sectional study | Lynch Syndrome patients, ≥ 18 years; n=55 | 55 | Facebook | NA | NR | I; F | NR | 45 D |
| Akard et al. 2015 [93] (USA) | Cross-sectional study | Parents or legal guardian caregivers of children (7 - 17 years) diagnosed with cancer; n= 45 | 45 | Facebook | NA | Paid | I; NR | NR | 10.5 WK |
| Amerson. 2011[103] (USA) | Web-based survey | Former nursing students; n=19 located | 19 | Facebook | NA | NR | NR; NR | NR | NR |
| Teo et al,74  2018 [73] (USA) | Web-based survey | Military veterans, ≥ 18 years; n=589 | 589 | Facebook | NA | Paid | I; F | NR | 45 D |
| Wilson & Usher. 2017 [108] (Australia) | Mixed method study | ˃ 18years; n= 81 | 81 | Twitter | NA | NR | NR; NR | NR | NR |
| Paige & Krieger. 2019 [66] (USA) | Web-based survey | Patients diagnosed with COPD; n=575 | 292 (51.0) | Social media | Research registry | NR | NR; F | NR | NR |

Multimedia Appendix 3. *(continued)*

| Author (Country) | Study design | Population &  Total n | Total n (%) enrolled/  Recruited through SM | SM platform | Other recruitment strategies | Ads paid/not paid | Incentive/ funded | Limitations of SM | Duration of ads |
| --- | --- | --- | --- | --- | --- | --- | --- | --- | --- |
| Child et al. 2014 [110] (USA) | Cross-sectional study | Emergency department registered nurses; n=190 | 78 | Facebook | NA | NR | I; NR | Inability to verify the authenticity of survey respondents or prevent multiply submissions. | NR |
| Ford et al. 2019 [47] (USA) | Cross-sectional study | Youths 13-20 years; n=828 | 828 | Facebook, Instagram, Snapchat | NA | Paid | I; NR | The anonymous link in the advertisement prevented identifying which social media platform yielded the most completed surveys and cost. | 48 D |
| Bennetts et al. 2019 [91] (Australia) | Secondary analysis | Employed parent 18-60 years; n= 4665 | 4665 | Facebook | NA | Paid | I; F | The Facebook algorithm and metrics keep changing. The self-selection by a participant in the study can be biased. | 15 WK |
| Wasilewski et al. 2019 [79] (Canada) | Secondary analysis | Family caregivers aged ≥18 years; n= 71 | 71 | Twitter | NA | NR | NR; NR | Twitter analytics tool failing to extract and analyze all study-related tweets. | 8 M |
| Bold et al. 2016 [30] (USA) | Web-based survey | Current adult smokers aged at least 18 years; n=272 | 272 | Facebook | NA | Paid | NI; F | Unable to determine sample representativeness recruited through Facebook. Self-reporting bias | 14 D |
| O’Connor et al. 2014 [104] (UK) | Cross-sectional study | Mothers of advanced maternal age ≥ 35 years; n=299 | 299 | Twitter | NA | NR | NR; NR | The personal information of users cannot be verified. | 11 WK |
| Arcia. 2014 [80] (USA) | Web-based survey | Nulliparous women: ≤ 20 weeks’ gestation. 18-44 years; n= 230 | 230 | Facebook | NA | Paid | I; F | The population of Facebook users is partially representative of the population hence findings cannot be generalized. Participants are not likely to complete demographic data | 18 WK |

Multimedia Appendix 3*. (continued)*

| Author (Country) | Study design | Population &  Total n | Total n (%) enrolled/  Recruited through SM | SM platform | Other recruitment strategies | Ads paid/not paid | Incentive/ funded | Limitations of SM | Duration of ads |
| --- | --- | --- | --- | --- | --- | --- | --- | --- | --- |
| Carter-Harris. 2016 [37] (USA) | Cross-sectional study | Current or former smokers aged 55 - 77 years; n=331 | 331 (100) | Facebook | NA | NR | NR; NR | Limited recruitment of a diverse population. Limits the participation of individuals without internet access | 18 D |
| Lohse. 2013 [62] (USA) | Web-based survey | Low-income women, 18-45 years; n=52 | 52 (100) | Facebook | NA | Paid | I; F | Participants provided self-reported responses that may be biased | 19 D |
| Choi et al. 2017 [40] (Australia) | Web-based survey | Males ≥ 18 years; n=398 | 398 (100) | Facebook | NA | Paid | NR; NR | The Facebook platform does not allow testing the impact of different ad images on recruitment rates and engagement. The Facebook algorithm prevented the interpretation and evaluation of study results | 4 WK |
| Chung et al. 2019 [111] (USA) | Cross-sectional study | Kidney transplant recipients, ≥ 18 years; n=153 | 153 (100) | Facebook | NA | Paid | I; NR | Social media recruitment generated a low response rate | 5 WK |
| Shaver et al. 2019 [71] (Canada) | Cross-sectional study | Adults aged 35-74 years; n=1048 | 1,048 (100) | Facebook | NA | Paid | NI; NF | The rural target ad limited the ability to conclude whether geographic targeting was effective. Facebook does not allow for the specification of age targets above 65 years. | 40 D |

Multimedia Appendix 3. *(continued)*

| Author (Country) | Study design | Population &  Total n | Total n enrolled/  Recruited through SM | SM platform | Other recruitment strategies | Ads paid/not paid | Incentive/ funded | Limitations of SM | Duration of ads |
| --- | --- | --- | --- | --- | --- | --- | --- | --- | --- |
| Gorman et al. 2014 [53] (USA) | Cohort study | Female AYA-aged cancer survivors, 16-35 years; n=534 | 387  (72.5) | Facebook, Craigslist, Twitter | Outreach, referrals, healthcare providers, clinics, community, word-of-mouth, information hotline | NR | NR; F | NR | NR |
| Wilkerson et al. 2015 [83] (USA) | Cross-sectional study | Methamphetamine-using MSM, aged ≥ 18 years; n=343 | 320 (93.3) | Facebook, Twitter | Ads on mobile websites, websites browsers | Paid | I; F | NR | NR |
| Herbell 2019 [115] (USA) | Secondary analysis | Pregnant women in the second or third trimester, at least 18 years. | 62 (76.0) | Facebook | Traditional methods | NR | NR; NR | NR | SM- 48 D; OS- 75 D |
|  |  |  |  |  |  |  |  |  |  |
| Iott et al. 2018 [57] (USA) | Cross-sectional study | MSM ≥ 18 years, self-identified males; n= 64 | 38 (59.3) | Facebook, Instagram, Grindr, Twitter | Newspaper articles, bar outreach, personal networking, Scruff ads, flyers, emails, staff recruitment, business locations | Paid | NR; F | NR | SM- 3 D; OS- NR |
| Akers & Gordon. 2018 [60] (USA) | Randomized controlled trial | Women, aged 25 - 65years and above; n=1145 randomized | 1,145 | Facebook | NA | Paid | I; F | NR | 91 D |
| Johnson et al. 2019 [2] (USA) | Web-based survey | Bereaved individuals; n=27 | 27 (100) | Facebook, Twitter | NA | NR | NR; NR | NR | 2 WK& 4 WK |

Multimedia Appendix 3. *(continued)*

| Author (country) | Study design | Population  & Total n | Total n (%) enrolled  /Recruited through SM | SM  Platform | Other  recruitment  Strategies | Ads paid/  Not paid | Incentive/ funded | Limitations of SM | Duration of ads |
| --- | --- | --- | --- | --- | --- | --- | --- | --- | --- |
| Pozzar et al. 2020 [68] (USA) | Cross-sectional study | Women diagnosed with ovarian cancer | 271 (100) | Facebook, Twitter | NA | NR | I; F | Internet Protocol address of respondent not collected | NR |
| Chu & Snider. 2013 [41] (Canada) | Secondary analysis | 15-24 years; n= 88 | 88 (100) | Facebook | NA | Paid | NR; NF | Exclusion of participants of lower socioeconomic status due to limited internet access. Self-selection bias as participants who chose to participate may have characteristics different than those who chose not to response. | NR |
| Stokes et  al. 2019 [105] (Canada) | Cross-sectional study | Registered nurses; n=267 | 267 (100) | Facebook, LinkedIn | NA | NP | NR; F | NR | 35 D |
| Kapp et al. 2013 [59] (USA) | Web-based survey | Women 35-49 years; n=9 | 9 (100) | Facebook | NA | Paid | I; F | NR | 10 D |
| Burke-Garcia & Mathew. 2017 [32] (USA) | Web-based survey | Parents of infants; n=1020 | NR | Facebook, Twitter, blogs, | Outreach, message boards | Paid | NR; F | NR | SM- 65 W |
| McCarthy & Mazza. 2019 [63] | Web-based survey | Women, 16-25 years; n=437 | 437 (100) | Facebook | NA | Paid | I; F | NR | 19 D |

Multimedia Appendix 3. *(continued)*

| Author (country) | Study design | Population  & Total n | Total n (%) enrolled  /Recruited through SM | SM  platform | Other  recruitment  Strategies | Ads paid/  Not paid | Incentive/ funded | Limitations of SM | Duration of ads |
| --- | --- | --- | --- | --- | --- | --- | --- | --- | --- |
| Ryan et al.  2019 [69] (Australia) | Mixed method survey | Females and males between 18 and 65 years; n=34 | 34 | Facebook | NA | Paid | NR; NF | Participants recruited through Facebook yielded a sample biased population | 4 WK |
| Peñaherrera et al. 2015 [67] (Ecuador) | Cross-sectional study | Individuals between 55 and 65 years; n= 274 | 24 (9.0) | Social media | Traditional methods (radio, newspaper, television) | NR | NR; F | NR | NR |
| Jones et al. 2015 [102] (USA) | Clinical trial | Urban Black women at risk of HIV; 18 - 29 years; n=230 | 230 (100) | Facebook | NA | Paid | NR; F | NR | NR |
| Tsai et al.  2019 [75] (USA) | Web-based survey | Chinese, Korean, and Lantix cancer survivors, ≥ 18 years; n=105 | 105 (100) | Facebook | NA | Paid | I; F | Participants' information cannot be verified on Facebook platforms. | 48 D |
| Subasinghe et al. 2016 [72] (Australia) | Cross-sectional study ` | Females aged 18-25 years; n=919 | 919 (100) | Facebook | NA | Paid | NI; F | NR | 2 Y |
| Valdez et al. 2014 [76] (USA) | Qualitative study | Filipino-Americans. ≥  18 years; n=148 | 148 (100) | Facebook | NA | Paid | I; F | NR | NR |
| Carter-Harris et al. 2016 [117].  (USA) | Cross-sectional study | Tobacco smokers aged between 55-77; n=361 | 331(92) | Facebook | Newspaper ads | Paid | I; F | The recruitment efficacy comparisons were impacted due to different geographic locations. | SM – 18D  OM- 3D |

Multimedia Appendix 3. *(continued)*

| Author (country) | Study design | Population &  Total n | Total n (%) enrolled/  Recruited through SM | SM platform | Other  recruitment strategies | Ads paid/not paid | Incentive/ funded | Limitations of SM | Duration of ads |
| --- | --- | --- | --- | --- | --- | --- | --- | --- | --- |
| Schwinn et al. 2017 [120]. (USA) | Longitudinal study | Adolescent girls 13 or 14 years; n=797 | NR | Facebook | Mail | Paid | I; F | NR | NR |
| Leach et al. 2019[119]  Australia | Web-based survey | Working parents of children ≤ 18 years; n=1468 | NR | Facebook | NA | Paid | I; F | The Facebook algorithms does not allow for the comparison of advertisements.  When snowballing technique is used, sample representativeness is affected. | 1 WK |

Multimedia Appendix 3. *(continued)*

| Author  (Country) | Study design | Population &  Total n | Total n (%) enrolled/  Recruited through SM | SM platform | Other  recruitment strategies | Ads paid/not paid | Incentive/ funded | Limitations of SM | Duration of ads |
| --- | --- | --- | --- | --- | --- | --- | --- | --- | --- |
| Barney et al. 2021 [84] (USA) | Cross-sectional survey | Females between 15 &19 years; n= 636 | 518 (81.4) | Facebook, Instagram | In-person & clinic-based | Yes | I; F | The study limits the comparison of data of the different recruitment methods, given that only two social media platforms were engaged for the recruitment process. | SM – 146 D  OM – 293 D |
| Moseson et al 2021 [85] (USA) | Cross-sectional survey | Previously pregnant women aged 15-49; n=98 | 84 (86) | Facebook, Reddit | Google Ads | Yes | I; F | Facebook and Google Ads were facilitated by a social marketing professional whiles Reddit was managed by the study team | SM – 1 M  OM – 1 M |
| Turner-McGrievy et al 2021 [86] (USA) | Cross-sectional survey | Adult African-American; n=568 | 43 (8) | Social media post | TV interviews, radio ads, personal referrals, community events, work listservs | NP | NR; F | NR | NR |
| Salvy et al 2020 [20] (USA) | Randomized controlled trial | 18-35 years; n=79 | 27 (34) | Facebook | In-person, targeted mailings | Yes | NR; F | Social media was limited to paid ads without exploring other approaches. | SM – 3 M  OM – 14 M |
| Stuart & Moore [96] 2021 (USA) | Cross-sectional survey | Nurses; n=536 | NR | Facebook | Flyers, word of mouth, professional organization journal | Yes | I; F | Facebook’s method of recruitment may have excluded some potential participants. | SM – 7 M  OM – NR |

Multimedia Appendix 3. *(continued)*

| Author  (Country) | Study design | Population &  Total n | Total n (%) enrolled/  Recruited through SM | SM platform | Other  recruitment strategies | Ads paid/not paid | Incentive/ funded | Limitations of SM | Duration of ads |
| --- | --- | --- | --- | --- | --- | --- | --- | --- | --- |
| Cho et al 2021 [97] (USA) | Cross-sectional survey | Parents (18 years & above) with children with advanced cancer (7-17 years); n=73 screened for eligibility, N=150 child-parent dyads | (100) | Facebook | NA | Yes | I; F |  | SM – 3 YR |
| Avery-Desmarais et al 2022 [98] (USA) | Cross-sectional survey | Nurses; n=394 | 269 (68.3) | Facebook | Email, word of mouth | NR | I; F | Study participation was limited to potential participants with internet access | SM – 96 HR  OM – NR |
| Harfield et al 2021 [87] (Australia) | Cross-sectional survey | 16-29 years; n=2,724 | 2,002 (73.5) | Facebook, Instagram | Email | Yes | I; NR |  | SM – 6 WK |
| Lindsay et al 2021 [88] (USA) | Mixed method | Latino fathers ≥ 21 years with a child between 2 and 8 years or 11 and 19 years. n=113 | 4 (3.5%) | Facebook | On-site, in-person, community, sports, church events, snowballing, flyers | No | I; NRS |  |  |
| Smith et al 2021 [101] (USA) | Mixed method | Nurses; n=10 | 0 (0) | Social media | Personal outreach, public outreach | Yea |  |  |  |
| Spahrkas et al 2021 [89] (The Netherlands) | Secondary analysis | Cancer patients and survivors; n=755 | 755 (100) | Facebook | NA | Yes | NR; F | The study sample reached is limited to only one platform. | SM – 8 M |

Multimedia Appendix 3. *(continued)*

| Author  (Country) | Study design | Population &  Total n | Total n (%) enrolled/  Recruited through SM | SM platform | Other  recruitment strategies | Ads paid/not paid | Incentive/ funded | Limitations of SM | Duration of ads |
| --- | --- | --- | --- | --- | --- | --- | --- | --- | --- |
| Bethel et al. 2021 [99] (USA) | cross-sectional survey | Nurses; n=4,205 | 4117 (98) | Social media | hospital listservs, nursing organization listservs, conferences | yes | I; F | Limited record of time spent in the recruitment effort. | SM- 22 D; SM + OM- 4 M & 6 M |
| Leighton et al. 2021 [100] (USA) | Web-based survey | Student nurses; n=208 | 208 (100) | Twitter, LinkedIn |  | NR | NR; NR | NR | SM – 30 D |

*Notes:* Ads, advertisements; ASD, autism spectrum disorder; AYA, adolescent and young adult ;COPD, chronic obstructive pulmonary disease; D, days; F, funded; I, incentive offered to participant; IRB, institutional research board; HR, hours; M, months; MSM, men who have sex with men; MSM-H, men who have sex with men-Hijras/transwomen; N, number; NA, not applicable; NR, not reported; NI, no incentive provided to participant; NF, not funded; NP, not paid; PLHIV, people living with HIV; SM, social media; OM, other media; OS, other strategies; YR, years; WK = week

Legend: Grey highlights are results from the updated search.
