# Supplementary material for: Social Media Use for Research Participant Recruitment: Integrative Literature Review
Source: J Med Internet Res. 2022 Aug 4;24(8):e38015. doi: 10.2196/38015 (PMC9389385; doi:10.2196/38015)
Supplement: Multimedia Appendix 4 [file jmir_v24i8e38015_app4.docx]

Multimedia Appendix 4: Overview of Quality Appraisal of Included Studies And CASP Checklist Used

| CASP Qualitative Studies Checklist | | | | | | | | | | |
| --- | --- | --- | --- | --- | --- | --- | --- | --- | --- | --- |
| Author/  Year | Was There A Clear Statement of The Aims Of The Research? | Is A Qualitative Methodology Appropriate? | Was The Research Design Appropriate To Address The Aims of The Research? | Was The Recruitment Strategy Appropriate To The Aims of The Research? | Was The Data Collected In A Way That Addressed The Research Issue? | Has The Relationship Between Researcher And Participants Been Adequately Considered? | Have Ethical Issues Been Taken Into Consideration? | Was The Data Analysis Sufficiently Rigorous? | Is There A Clear Statement Of Findings? | Overall Quality Assessment |
| Chambers et al. 2020 109] | Y | Y | Y | Y | Y | N | N | N | Y | M |
| Valdez et al. 2014 [76] | Y | Y | Y | Y | Y | Y | Y | Y | Y | H |
| Hansen et al. 2016 [114] | Y | Y | Y | Y | Y | Y | Y | N | Y | M |

| Multimedia Appendix 4. CASP Cohort Studies Checklist | | | | | | | | | | | | | | |
| --- | --- | --- | --- | --- | --- | --- | --- | --- | --- | --- | --- | --- | --- | --- |
| Author/ Year | Did The Study Address A Clearly Focused Issue? | Was The Cohort Recruited In An Acceptable Way? | Was The Exposure Accurately Measured To Minimize Bias? | Was The Outcome Accurately Measured To Minimize Bias? | Have The Authors Identified All Important Confounding Factors? | Have They Taken Account Of The Confounding Factors In The Design And/or Analysis? | Was The Follow Up Of Subjects Complete Enough? | Was The Follow Up Of Subjects Long Enough? | Do You Believe The Results? | Can The Results Be Applied To The Local Population? | Do The Results Of This Study Fit With Other Available Evidence? | What Are The Implications Of This Study For Practice? | Overall Quality Assessment |  |
| Gorman et al. 2014 [53] | Y | Y | Y | N | N | C | Y | N | Y | Y | Y | Y | M |  |
| van Gelder et al. 2019 [77] | Y | Y | N | C | Y | Y | Y | Y | Y | Y | Y | Y | M |  |
| Harris et al. 2015 [55] | Y | Y | Y | Y | Y | Y | Y | Y | Y | Y | Y | Y | M |  |

Multimedia Appendix 4*.* CASP Randomized Controlled Trial Standard Checklist

|  | | | | | | | | | | | | | |
| --- | --- | --- | --- | --- | --- | --- | --- | --- | --- | --- | --- | --- | --- |
| Author/ Year | Did The Study Address A Clearly Focused Research Question? | Was The Assignment of Participants To Interventions Randomized? | Were All Participants Who Entered The Study Accounted For At Its Conclusion? | Were The Participants ‘Blind’ To Intervention They Were Given? | Were The Study Groups Similar At The Start Of The Randomized Controlled Trial? | Apart From The Experimental Intervention, Did Each Study Group Receive The Same Level of Care | Were The Effects Of Intervention Reported Comprehensively? | Was The Precision of The Estimate Of The Intervention Or Treatment Effect Reported? | Do The Benefits of The Experimental Intervention Outweigh The Harms And Costs? | Can The Results Be Applied To Your Local Population/In Your Context? | Would The Experimental Intervention Provide Greater Value To The People In Your Care Than Any Of The Existing Interventions? | Overall Quality Assessment |  |
| Frandsen et al. 2014 [50] | N | Y | Y | C | Y | Y | N | N | C | Y | Y | M |  |
| Juraschek et al. 2018 [58] | N | C | C | C | Y | Y | Y | N | C | Y | Y | L |  |
| Watson et al. 2018 [81] | N | Y | C | C | Y | Y | Y | C | C | Y | Y | M |  |
| Burgess et al. 2017 [31] | N | Y | N | C | Y | Y | N | N | C | Y | Y | M |  |
| Kelleher et al. 2018 [61] | N | Y | Y | Y | Y | Y | Y | N | C | Y | Y | M |  |

Multimedia Appendix 4. CASP Randomized Controlled Trial Standard Checklist

| Author/ Year | Did The Study Address A Clearly Focused Research Question? | Was The Assignment of Participants To Interventions Randomized? | Were All Participants Who Entered The Study Accounted For At Its Conclusion? | Were The Participants ‘Blind’ To Intervention They Were Given? | Were The Study Groups Similar At The Start Of The Randomized Controlled Trial? | Apart From The Experimental Intervention, Did Each Study Group Receive The Same Level of Care | Were The Effects Of Intervention Reported Comprehensively? | Was The Precision of The Estimate Of The Intervention Or Treatment Effect Reported? | Do The Benefits of The Experimental Intervention Outweigh The Harms And Costs? | Can The Results Be Applied To Your Local Population/In Your Context? | Would The Experimental Intervention Provide Greater Value To The People In Your Care Than Any Of The Existing Interventions? | Overall Quality Assessment |
| --- | --- | --- | --- | --- | --- | --- | --- | --- | --- | --- | --- | --- |
| Guthrie et al. 2019 [54] | N | Y | C | C | Y | C | N | N | C | Y | Y | L |
| Akers & Gordon. 2018 [60] | N | Y | Y | C | Y | Y | Y | N | C | Y | Y | M |
| Moreno et al. 2017 [18] | N | Y | Y | C | Y | Y | Y | N | C | Y | Y | M |
| Waltman et al. 2020 [106] | N | Y | Y | C | Y | Y | N | N | C | Y | Y | M |
| Salvy et al. 2020 [20] | N | Y | Y | C | Y | Y | N | N | C | Y | Y | M |
|  |  |  |  |  |  |  |  |  |  |  |  |  |

*Notes.* The following gradings were used according to the CASP checklist guidelines: Y= yes; C= Can´t tell; N
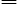
No. *H*=high overall quality, M = moderate overall quality, L = low overall quality.

Legend: Grey highlight is the result from the updated search.
