# Supplementary material for: Social Media Use for Research Participant Recruitment: Integrative Literature Review
Source: J Med Internet Res. 2022 Aug 4;24(8):e38015. doi: 10.2196/38015 (PMC9389385; doi:10.2196/38015)
Supplement: Multimedia Appendix 5 [file jmir_v24i8e38015_app5.docx]

Multimedia Appendix 5. List of Excluded Studies (n=104)

1. Adrian M, Moreno M, Nicodimos S, McCauley E, Stoep AV. Research strategy for health sciences: Facebook friend request is non-differentially accepted in a diverse, young adult population. *Nurs Health Sci*. 2019;21(1):71-77. doi:10.1111/nhs.12557

***Reason for exclusion: Wrong concept***

1. Akard TF. Facebook advertising for pediatric oncology research recruitment (FR461C). *J Pain Symptom Manage*. 2016;51(2):373. doi:10.1016/j.jpainsymman.2015.12.238

***Reason for exclusion: Duplicate***

1. Akard TF. Facebook advertising for pediatric oncology research recruitment (FR461C). *J Pain Symptom Manage*. 2016;51(2):373. doi:10.1016/j.jpainsymman.2015.12.238

***Reason for exclusion: Non-research***

1. Akard TF, Wray S, Gilmer MJ. Facebook advertisements recruit parents of children with cancer for an online survey of web-based research preferences. *Cancer Nurs*. 2015;38(2):155-161. doi:10.1097/NCC.0000000000000146

***Reason for exclusion: Duplicate***

1. Foster Akard T, Gerhardt CA, Hendricks-Ferguson V, et al. Facebook advertising to recruit pediatric populations. *J Palliat Med*. 2016;19(7):692-693. doi:10.1089/jpm.2016.0128

***Reason for exclusion: Non-research***

1. Allsworth JE. Invited commentary: Recruiting for epidemiologic studies using social media. *Am J Epidemiol*. 2015;181(10):747-749. doi:10.1093/aje/kwv007

***Reason for exclusion: Non-research***

1. Alto KM, McCullough KM, Levant RF. Who is on Craigslist? A novel approach to participant recruitment for masculinities scholarship. *Psychol Men Masculinity*. 2018;19(2):319-324. doi:10.1037/men0000092

***Reason for exclusion: Non-health discipline***

1. Amon KL, Campbell AJ, Hawke C, Steinbeck K. Facebook as a recruitment tool for adolescent health research: a systematic review. *Acad Pediatr*. 2014;14(5):439-447.e4. doi:10.1016/j.acap.2014.05.049

***Reason for exclusion: Review***

1. Bamidele OO, E. McGarvey H, Lagan BM, Chinegwundoh F, Ali N, McCaughan E. “Hard to reach, but not out of reach”: barriers and facilitators to recruiting black African and black Caribbean men with prostate cancer and their partners into qualitative research. *Eur J Cancer Care (Engl)*. 2019;28(2):1-11. doi:10.1111/ecc.12977

***Reason for exclusion: Wrong concept***

1. Baker R, Zlotorzynska M, Knopf AS. Use of social media for the recruitment and engagement of adolescent sexual and gender minorities in HIV research studies. *J Adolesc Health*. 2020;66(2):S39-S40. doi:10.1016/j.jadohealth.2019.11.079

***Reason for exclusion: Non-research***

1. Beattie E, Fielding E, O’Reilly M, Brooks D, MacAndrew M, McCrow J. Recruitment of individuals with dementia and their carers for social research: lessons learned from nine studies. *Res Gerontol Nurs*. 2018;11(3):119-128. doi:10.3928/19404921-20180308-01

***Reason for exclusion: Wrong concept***

1. Biedermann N. The use of Facebook for virtual asynchronous focus groups in qualitative research. *Contemp Nurse*. 2018;54(1):26-34. doi:10.1080/10376178.2017.1386072

***Reason for exclusion: Wrong concept***

1. Bowen A, Williams M, Horvath K. Using the internet to recruit rural MSM for HIV risk assessment: sampling issues. *AIDS Behav*. 2004;8(3):311-319. doi:10.1023/B:AIBE.0000044078.43476.1f

***Reason for exclusion: Wrong concept***

1. Bramstedt KA. Recruiting healthy volunteers for research participation via internet advertising. *Clin Med Res*. 2007;5(2):91-97. doi:10.3121/cmr.2007.718

***Reason for exclusion: Wrong concept***

1. Bushar JA, Fishman J, Garfinkel D, Pirretti A. Enrolling underserved women in mHealth programs: results from text4baby outreach campaigns. *Health Promot Pract*. 2019;20(2):292-299. doi:10.1177/1524839918763589

***Reason for exclusion: Wrong concept***

1. Cahill TJ, Wertz B, Zhong Q, et al. Correction: The search for consumers of web-based raw DNA interpretation services: using social media to target hard-to-reach populations. *J Med Internet Res*. 2019;21(8):e15735. doi:10.2196/15735

***Reason for exclusion: Duplicate***

1. Carlson RH. Internet as recruiting station for clinical cancer trials. *Oncol Times*. 2004;26(21):37. doi:10.1097/01.cot.0000314426.99168.ac

***Reason for exclusion: Non-research***

1. Chinnery F, Francis N, Griffiths G, et al. SWAT: Can social media advertising enhance trial recruitment? Trials. 2019;20(S1):129. http://dx.doi.org/10.1186/s13063-019-3688-6

***Reason for exclusion: Record not available***

1. Chow A, Mitter N, Bong YL, et al. Social media for research recruitment. *Annals of the Acad of Med.* 2010;*39*(11):S319. <http://www.annals.edu.sg/pdf/39VolNo11SupplNov2010/V39N11(Suppl).pdf>

***Reason for exclusion: Record not available***

1. Comabella C, Wanat M. The use of social media to recruit participants in cancer care research: challenges and opportunities. *Psycho-Oncol*. 2013;22(3),163. http://doi.org/10.1111/j.1099-1611.2013.3394

***Reason for exclusion: Record not available***

1. Cowie JM, Gurney ME. The use of Facebook advertising to recruit healthy elderly people for a clinical trial: baseline metrics. *JMIR Res Protoc*. 2018;7(1):e20. doi:10.2196/resprot.7918

***Reason for exclusion: Duplicate***

1. Cudjoe J, Turkson-Ocran RA, Ezeigwe AK, Commodore-Mensah Y, Nkimbeng M, Han HR. Recruiting African immigrant women for community-based cancer prevention studies: lessons learned from the AfroPap study. *J Community Health*. 2019;44(5):1019-1026. doi:10.1007/s10900-019-00677-y

***Reason for exclusion: Wrong concept***

1. Denhoff ER, Milliren CE, De Ferranti SD, Steltz SK, Osganian SK. Factors associated with clinical research recruitment in a pediatric academic medical center-a web-based survey. *PLoS One*. 2015;10(10):1-19. doi:10.1371/journal.pone.0140768

***Reason for exclusion: Wrong concept***

1. Eagan SM, Johnson EK, Eagan LXN. A paradox of choice and opportunity in the social mediated participant recruitment space: opportunities and caveats. *Am J Bioeth*. 2019;19(6):76-78. doi:10.1080/15265161.2019.1602178

***Reason for exclusion: Non-health discipline***

1. Ehrenberger HE. The e-recruitment of participants for clinical trials. *IRB*. 2003;25(4):16-17. doi:10.2307/3563820

***Reason for exclusion: Wrong concept***

1. Elkin PL, Schlegel DR, Anand E. Recruiting participants to local clinical trials using ontology and the IoT. *Stud Health Technol Inform*. 2016;221:119. doi:10.3233/978-1-61499-633-0-119

***Reason for exclusion: Wrong concept***

1. Etkin CD, Farran CJ, Barnes LL, Shah RC. Recruitment and enrollment of caregivers for a lifestyle physical activity clinical trial. *Res Nurs Heal*. 2012;35(1):70-81. doi:10.1002/nur.20466

***Reason for exclusion: Wrong concept***

1. Im EO, Lee Y, Ji X, et al. Internet recruitment of Asian American breast cancer survivors. *ANS Adv Nurs Sci*. 2016;39(3):E17-E27. doi:10.1097/ans.0000000000000131

***Reason for exclusion: Wrong concept***

1. Ferrigno C, He J, Cordova CP, Fair J, Shakoor N, Wimmer MA. Exploring the use of online social media advertising to recruit patients in an osteoarthritis clinical trial. *Osteoarthr Cartil*. 2018;26(2018):S271-S272. doi:10.1016/j.joca.2018.02.552

***Reason for exclusion: Non-research***

1. Ferrigno BN, Sade RM. Ethics of recruiting research subjects through social media. *Am J Bioeth*. 2019;19(6):73-75. doi:10.1080/15265161.2019.1602192

***Reason for exclusion: Non-research***

1. Fernandez MI, Warren JC, Varga LM, Prado G, Hernandez N, Bowen GS. Cruising in cyber space: comparing internet chat room versus community venues for recruiting hispanic men who have sex with men to participate in prevention studies. *J Ethn Subst Abuse*. 2007;6(2):143-162. doi:10.1300/J233v06n02_09

***Reason for exclusion: Wrong concept***

1. Frampton GK, Shepherd J, Pickett K, Griffiths G, Wyatt JC. Digital tools for the recruitment and retention of participants in randomised controlled trials: a systematic map. *Trials*. 2020;21(1):1-24. doi:10.1186/s13063-020-04358-3

***Reason for exclusion: Review***

1. Freeman HP. Considerations on the use of the internet as a tool for minority recruitment into clinical trials. *Cancer J*. 2006;12(6):459-460. doi:10.1097/00130404-200611000-00004

***Reason for exclusion: Non-research***

1. Gabrielli J, Borodovsky J, Corcoran E, Sink L. Leveraging social media to rapidly recruit a sample of young adults aging out of foster care: methods and recommendations. *Child Youth Serv Rev*. 2020;113(December 2019):104960. doi:10.1016/j.childyouth.2020.104960

***Reason for exclusion: Non-health discipline***

1. Gelinas L, Pierce R, Winkler S, Cohen G, Lynch HF, Bierer BE. Nonexceptionalism, research risks, and social media: response to open peer commentaries on “using social media as a research recruitment tool: ethical issues and recommendations.” *Am J Bioeth*. 2017;17(5):W1-W3. doi:10.1080/15265161.2017.1293755

***Reason for exclusion: Non-research***

1. Gelinas L, Pierce R, Winkler S, Cohen G, Lynch HF, Bierer BE. Nonexceptionalism, research risks, and social media: response to open peer commentaries on “using social media as a research recruitment tool: ethical issues and recommendations.” *Am J Bioeth*. 2017;17(5):W1-W3. doi:10.1080/15265161.2017.1293755

***Reason for exclusion: Duplicate***

1. Giustini D. Social media and clinical trials recruitment: potential benefits and challenges. *J Can Heal Libr Assoc / J l’Association des bibliothèques la santé du Canada*. 2014;33(3):140. doi:10.5596/c12-044

***Reason for exclusion: Non-research***

1. Gross MS, Liu NH, Contreras O, Muñoz RF, Leykin Y. Using Google Adwords for international multilingual recruitment to health research websites. *J Med Internet Res*. 2014;16(1):1-11. doi:10.2196/jmir.2986

***Reason for exclusion: Wrong concept***

1. Guevara E. Recruiting ethnic minority cancer patients for internet research. *Southern Online Journal of Nursing Research*. 2008;8(2)

***Reason for exclusion: Non-research***

1. Guthrie K, Caan B, Diem S, Ensrud K, Greaves S, Lacroix A. *Comparison of* Mass Mailings to Facebook Advertising for Recruitment of Midlife Women with Bothersome Vaginal Symptoms*. Clin Trials*. 2018;15(2_suppl):S35-S192. doi:10.1177/1740774518790846

***Reason for exclusion: Non-research***

1. Hamilton RJ, Bowers BJ. Internet recruitment and e-mail interviews in qualitative studies. *Qual Health Res*. 2006;16(6):821-835. doi:10.1177/1049732306287599

***Reason for exclusion: Wrong concept***

1. Holland CM, Ritchie ND, Du Bois SN. iTunes Song-Gifting is a Low-Cost, Efficient Recruitment Tool to Engage High-Risk MSM in Internet Research. *AIDS Behav*. 2015;19(10):1914-1918. doi:10.1007/s10461-015-1130-y

***Reason for exclusion: Wrong concept***

1. Hugelius K, Adolfsson A, Gifford M, Örtenwall P. Facebook enables disaster research studies: the use of social media to recruit participants in a post-disaster setting. *PLoS Curr*. 2016;8(Disasters):1-11. doi:10.1371/currents.dis.f4a444e1f182776bdf567893761f86b8

***Reason for exclusion: Non-health discipline***

1. Hugelius K, Adolfsson A, Gifford M, Örtenwall P. Facebook enables disaster research studies: the use of social media to recruit participants in a post-disaster setting. *PLoS Curr*. 2016;8(Disasters):1-11. doi:10.1371/currents.dis.f4a444e1f182776bdf567893761f86b8

***Reason for exclusion: Duplicate***

1. Im EO, Chee W, Lim HJ, et al. Recruitment of oncology nurses for internet research: issues and future directions. *Oncol Nurs Forum*. 2006;33(2):249-254. doi:10.1188/06.ONF.249-254

***Reason for exclusion: Wrong concept***

1. Im EO, Chee W. Recruitment of research participants through the Internet. *Comput Inform Nurs*. 2004;22(5):289-297. doi:10.1097/00024665-200409000-00009

***Reason for exclusion: Wrong concept***

1. Jenkins GP, Aronsky D. A feasibility study for the computerized recruitment of subjects for research studies. *AMIA Annu Symp Proc*. Published online 2005:996.

***Reason for exclusion: Wrong concept***

1. Jones J, Salazar LF. A review of HIV prevention studies that use social networking sites: implications for recruitment, health promotion campaigns, and efficacy trials. *AIDS Behav*. 2016;20(11):2772-2781. doi:10.1007/s10461-016-1342-9

***Reason for exclusion: Review***

1. Kelleher E, Moreno M, Wilt MP. Recruitment of participants and delivery of online mental health resources for depressed individuals using Tumblr: pilot randomized control trial. *JMIR Res Protoc*. 2018;7(4):e95. doi:10.2196/resprot.9421

***Reason for exclusion: Duplicate***

1. King DB, O’Rourke N, De Longis A. Social media recruitment and online data collection: a beginner’s guide and best practices for accessing low-prevalence and hard-to-reach populations. *Can Psychol*. 2014;55(4):240-249. doi:10.1037/a0038087

***Reason for exclusion: Non-health discipline***

1. Koenings M, Martin-Biggers J, Byrd-Bredbenner C. Recruiting RCT participants: To Facebook or not to Facebook? *FASEB J*. 2015;29(S1). doi:10.1096/fasebj.29.1_supplement.584.2

***Reason for exclusion: Non-research***

1. Komotar RJ, Zacharia BE, Mocco J, et al. Internet-based approach to multi-institutional patient recruitment in the medico-legal age. *J Clin Oncol*. 2005;23(19):4471-4473. doi:10.1200/JCO.2005.01.5271

***Reason for exclusion: Non-research***

1. Langbaum JB, High N, Nichols J, Kettenhoven C, Reiman EM, Tariot PN. The Alzheimer’s prevention registry: a large internet-based participant recruitment registry to accelerate referrals to Alzheimer’s focused studies. *J Prev Alzheimer’s Dis*. 2020;7(4):242-250. doi:10.14283/jpad.2020.31

***Reason for exclusion: Wrong concept***

1. Logsdon MC, Rushton J, Myers J, Gregg J, Bennett G. Use of social media for research recruitment With adolescent mothers. *J Adolesc Health*. 2015;56(2):S21-S22. doi:10.1016/j.jadohealth.2014.10.043

***Reason for exclusion: Non-research***

1. Logsdon MC, Rushton J, Myers J, Gregg J, Bennett G. Use of social media for research recruitment With adolescent mothers. *J Adolesc Health*. 2015;56(2):S21-S22. doi:10.1016/j.jadohealth.2014.10.043

***Reason for exclusion: Non-research***

1. Mailey KA, O’leary DE, Percer P, Nihira MA. Women over 50 use social media! A potential tool for subject recruitment and patient education. *Female Pelvic Med Reconstr Surg*. 2015;21(5):S55-S146. doi:10.1097/spv.0000000000000207

***Reason for exclusion: Record not available***

1. Maloni JA, Przeworski A, Damato EG. Web recruitment and internet use and preferences reported by women with postpartum depression after pregnancy complications. *Arch Psychiatr Nurs*. 2013;27(2):90-95. doi:10.1016/j.apnu.2012.12.001

***Reason for exclusion: Wrong concept***

1. Marks A, Wilkes L, Blythe S, Griffiths R. A novice researcher’s reflection on recruiting participants for qualitative research. *Nurse Res*. 2017;25(2):34-38. doi:10.7748/nr.2017.e1510

***Reason for exclusion: Non-research***

1. McAloney-Kocaman K, Lorimer K, Flowers P, Davis M, Knussen C, Frankis J. Sexual identities and sexual health within the Celtic nations: an exploratory study of men who have sex with men recruited through social media. *Glob Public Health*. 2016;11(7-8):1049-1059. doi:10.1080/17441692.2016.1185450

***Reason for exclusion: Wrong concept***

1. Mendelson C. Recruiting participants for research from online communities. *CIN - Comput Informatics Nurs*. 2007;25(6):317-323. doi:10.1097/01.NCN.0000299653.13777.51

***Reason for exclusion: Wrong concept***

1. Odwazny LM. The “nonexceptionalism” of social media used for subject recruitment. *Am J Bioeth*. 2017;17(3):17-19. doi:10.1080/15265161.2016.1274803

***Reason for exclusion: Non-research***

1. Pandya LK, Nekkanti S, Hundley AF, Hudson CO, Lynch CD. Social networking to access patients (SNAP): Utilizing Facebook as a recruitment tool for research in pelvic floor disorders. *Female Pelvic Med Reconstr Surg*. 2017;23(5S):S76-S137. doi:10.1097/spv.0000000000000479

***Reason for exclusion: Non-research***

1. Pandya LK, Nekkanti S, Hundley A, Hudson C, Lynch C. 42: Social networking to access patients (SNAP): utilizing internet-based platforms as a recruitment tool for research in pelvic floor disorders. *Am J Obstet Gynecol*. 2018;218(2):S920-S921. doi:10.1016/j.ajog.2017.12.061

***Reason for exclusion: Non-research***

1. Park BK, Calamaro C. A systematic review of social networking sites: innovative platforms for health research targeting adolescents and young adults. *J Nurs Scholarsh*. 2013;45(3):256-264. doi:10.1111/jnu.12032

***Reason for exclusion: Review***

1. Pedersen ER, Kurz J. Using Facebook for health-related research study recruitment and program delivery. *Curr Opin Psychol*. 2016;9:38-43. doi:10.1016/j.copsyc.2015.09.011

***Reason for exclusion: Review***

1. Penarobichaux V, Watson AJ, Kvedar JC. The evaluation of online tools in the recruitment of dermatology patients for a research study. *J Am Acad Dermatol*. 2010;62(3):AB64. doi:10.1016/j.jaad.2009.11.266

***Reason for exclusion: Non-research***

1. Pena-Robichaux V, Watson AJ. Recruiting subjects for research studies in the era of web 2.0. *J Am Acad Dermatol*. 2010;63(3):e69-e70. doi:10.1016/j.jaad.2009.12.021

***Reason for exclusion: Non-research***

1. Porucznik CA, Schliep KC, Stanford JB. Participant recruitment in a virtually interconnected world: Comparative effectiveness and considerations of bias. *American Journal of Epidemiology*. 2010;171(S11):S1-S157. doi:10.1093/aje/kwq151

***Reason for exclusion: Non-research***

1. Rae K, Weatherall L, Hollebone K, et al. Developing research in partnership with Aboriginal communities - strategies for improving recruitment and retention. *Rural Remote Health*. 2013;13(1):1-8. doi:10.22605/rrh2255

***Reason for exclusion: Non-research***

1. Reagan L, Nowlin SY, Birdsall SB, et al. Integrative review of recruitment of research participants through Facebook. *Nurs Res*. 2019;68(6):423-432. doi:10.1097/NNR.0000000000000385

***Reason for exclusion: Review***

1. Reagan L, Nowlin SY, Birdsall SB, et al. integrative review of recruitment of research participants through Facebook. *Nurs Res*. 2019;68(6):423-432. doi:10.1097/NNR.0000000000000385

***Reason for exclusion: Duplicate***

1. Reaves AC, Bianchi DW. The role of social networking sites in medical genetics research. *Am J Med Genet Part A*. 2013;161(5):951-957. doi:10.1002/ajmg.a.35903

***Reason for exclusion: Review***

1. Reaves AC, Bianchi DW. The role of social networking sites in medical genetics research. *Am J Med Genet Part A*. 2013;161(5):951-957. doi:10.1002/ajmg.a.35903

***Reason for exclusion: Duplicate***

1. Reed E, Simmonds P, Corner J. Surveying the experience of living with metastatic breast cancer: comparing face-to-face and online recruitment. *J Res Nurs*. 2009;14(1):43-55. doi:10.1177/1744987108100013

***Reason for exclusion: Wrong concept***

1. Refolo P, Sacchini D, Minacori R, Daloiso V, Spagnolo AG. E-recruitment based clinical research: notes for research ethics committees/institutional review boards. *Eur Rev Med Pharmacol Sci*. 2015;19(5):800-804

***Reason for exclusion: Wrong concept***

1. Reynolds T. Clinical trials: Can technology solve the problem of low recruitment? *BMJ*. 2011;342(7811):1338-1339. doi:10.1136/bmj.d3662

***Reason for exclusion: Non-research***

1. Richiardi L, Pivetta E, Merletti F. Recruiting study participants through Facebook. *Epidemiology*. 2012;23(1):175. doi:10.1097/EDE.0b013e31823b5ee4

***Reason for exclusion: Non-research***

1. Rife SC, Cate KL, Kosinski M, Stillwell D. Participant recruitment and data collection through Facebook: the role of personality factors. *Int J Soc Res Methodol*. 2016;19(1):69-83. doi:10.1080/13645579.2014.957069

***Reason for exclusion: Non-health discipline***

1. Rodriguez C, Sanchez C, Grzenda A, et al. The use of social media in recruiting participants for mental health research purposes: A systematic review and recommendations. *Neuropsychopharmacology*. 2019;44(S1):230-384. doi:10.1038/s41386-019-0546-x

***Reason for exclusion: Review***

1. Ryan GS. Online social networks for patient involvement and recruitment in clinical research. *Nurse Res*. 2013;21(1):35-39. doi:10.7748/nr2013.09.21.1.35.e302

***Reason for exclusion: Review***

1. Samuel G. “The danger of lurking”: different conceptualizations of “user awareness” in social media research. *Am J Bioeth*. 2017;17(3):25-26. doi:10.1080/15265161.2016.1274793

***Reason for exclusion: Non-research***

1. Sanchez C, Grzenda A, Varias A, et al. Social media recruitment for mental health research: a systematic review. *Compr Psychiatry*. 2020;103:152197. doi:10.1016/j.comppsych.2020.152197

***Reason for exclusion: Review***

1. Schlupp A, Castillo M, Vickroy W, Lopez A, Sanders RA. Reaching and recruiting young black and Latino MSM: understanding effective social media campaigns for youth-focused research. *J Adolesc Heal*. 2020;66(2):S128-S129. doi:10.1016/j.jadohealth.2019.11.257

***Reason for exclusion: Non-research***

1. Schumacher KR, Lee JM. Harnessing social media for child health research pediatric research 2.0. *JAMA Pediatr*. 2016;170(1):5-6. doi:10.1001/jamapediatrics.2015.2696

***Reason for exclusion: Non-research***

1. Sedrak MS, Sun V, Liu J, et al. Physician perceptions of the use of social media for recruitment of patients in cancer clinical trials. *JAMA Netw Open*. 2019;2(9):1-10. doi:10.1001/jamanetworkopen.2019.11528

***Reason for exclusion: Wrong concept***

1. Seltzer ED, Stolley MR, Mensah EK, Sharp LK. Social networking site usage among childhood cancer survivors-a potential tool for research recruitment? *J Cancer Surviv*. 2014;8(3):349-354. doi:10.1007/s11764-014-0348-4

***Reason for exclusion: Wrong concept***

1. Seltzer ED, Stolley MR, Mensah EK, Sharp LK. Social networking site usage among childhood cancer survivors-a potential tool for research recruitment? *J Cancer Surviv*. 2014;8(3):349-354. doi:10.1007/s11764-014-0348-4

***Reason for exclusion: Duplicate***

1. Shatz I. Fast, free, and targeted: Reddit as a source for recruiting participants online. *Soc Sci Comput Rev*. 2017;35(4):537-549. doi:10.1177/0894439316650163

***Reason for exclusion: Non-health discipline***

1. Sikkens E, van San M, Sieckelinck S, Boeije H, de Winter M. Participant Recruitment through Social Media: Lessons Learned from a Qualitative Radicalization Study Using Facebook. *Field methods*. 2017;29(2):130-139. doi:10.1177/1525822X16663146

***Reason for exclusion: Non-health discipline***

1. Spence PR, Lachlan KA, Rainear AM. Social media and crisis research: data collection and directions. *Comput Human Behav*. 2016;54:667-672. doi:10.1016/j.chb.2015.08.045

***Reason for exclusion: Non-health discipline***

1. Shpilberg S. Social media for clinical trial patient. *Am Med Writ Assoc*. 2017;32(4):162-165.

***Reason for exclusion: Non-research***

1. Swirsky ES. A billion tiny ends: social media, nonexceptionalism, and ethics by association. *Am J Bioeth*. 2017;17(3):15-17. doi:10.1080/15265161.2016.1275883

***Reason for exclusion: Non-research***

1. Tebb KP, Schwarz EB, Rodriguez F, et al. Adapting to political barriers in conducting teen pregnancy prevention research: use of social media as an expedited recruitment strategy. *J Adolesc Heal*. 2020;66(2):S115. doi:10.1016/j.jadohealth.2019.11.230

***Reason for exclusion: Non-research***

1. Teo AR, Liebow SB, Chan B, Dobscha SK, Graham AL. Correction: Reaching those at risk for psychiatric disorders and suicidal ideation: Facebook advertisements to recruit military veterans. *JMIR Ment Health*. 2019;6(1):e13035. doi:10.2196/13035

***Reason for exclusion: Duplicate***

1. Ting TT, Chen CY, Tsai YS, Chen YT, Su LW, Chen WJ. Using social network as a recruiting tool for research on substance use in the Taipei metropolitan area: study design, implementation, and epidemiological estimates. *J Epidemiol*. 2015;25(10):647-655. doi:10.2188/jea.JE20140229

***Reason for exclusion: Wrong concept***

1. Topolovec-Vranic J, Natarajan K. The use of social media in recruitment for medical research studies: a scoping review. *J Med Internet Res*. 2016;18(11). doi:10.2196/jmir.5698

***Reason for exclusion: Review***

1. Tustin JL, Crowcroft NS, Gesink D, Johnson I, Keelan J, Lachapelle B. User-driven comments on a Facebook advertisement recruiting Canadian parents in a study on immunization: content analysis. *JMIR Public Heal Surveill*. 2018;4(9):1-12. doi:10.2196/10090

***Reason for exclusion: Wrong concept***

1. Tustin JL, Crowcroft NS, Gesink D, Johnson I, Keelan J, Lachapelle B. User-driven comments on a Facebook advertisement recruiting Canadian parents in a study on immunization: content analysis. *JMIR Public Health Surveill*. 2018;4(3):e10090. doi:10.2196/10090

***Reason for exclusion: Duplicate***

1. Verbrugge J, Rumbaugh M, Cook L, et al. The promise and pitfalls of Facebook advertising: a genetic counselor’s perspective. *J Genet Couns*. 2018;27(2):326-328. doi:10.1007/s10897-017-0207-3

***Reason for exclusion: Non-research***

100. Wamboldt P, Lohse B. Facebook successfully recruited low‐income participants to nutrition program evaluation. *FASEB J*. 2013;27(S1). doi:10.1096/fasebj.27.1_supplement.625.12

***Reason for exclusion: Record not available***

101. Whitaker C, Stevelink S, Fear N. The use of Facebook in recruiting participants for health research purposes: a systematic review. *J Med Internet Res*. 2017;19(8):1-11. doi:10.2196/jmir.7071

***Reason for exclusion: Review***

102. Worthen MGF. An invitation to use Craigslist ads to recruit respondents from stigmatized groups for qualitative interviews. *Qual Res*. 2014;14(3):371-383. doi:10.1177/1468794113481791

***Reason for exclusion: Non-health discipline***

103. Young SD, Harrell L, Jaganath D, Cohen AC, Shoptaw S. Feasibility of recruiting peer educators for an online social networking-based health intervention. *Health Educ J*. 2013;72(3):276-282. doi:10.1177/0017896912440768

***Reason for exclusion: Wrong concept***

104. Zamora TJ, Piazza C, Drobnich D, Amdur A, Headopohl W, Stepnowsky C. Using social media to recruit for sleep research studies. *SLEEP*. 2016;39:A393-A394.

***Reason for exclusion: Record not available.***

105. Kutok ER, Doria N, Dunsiger S, et al. Feasibility and cost of using instagram to recruit adolescents to a remote intervention. *J Adolesc Heal*. 2021;69(5):838-846. doi:10.1016/j.jadohealth.2021.04.021

***Reason for exclusion:*** Non-health discipline

106. Spellecy R, Nelson LD. How should investigators advertise on social media for research opportunities? *Am J Bioeth*. 2021;21(10):42-43. doi:10.1080/15265161.2021.1965254

***Reason for exclusion:*** Non-research

107. Flood-Grady E, Solberg LB, Baralt C, Meyer M, Stevens J, Krieger JL. Engaging institutional stakeholders to develop and implement guidelines for recruiting participants in research studies using social media: Mixed Methods, Multi-Phase Process. *J Med Internet Res*. 2021;23(10):N.PAG-N.PAG. doi:10.2196/23312

***Reason for exclusion:*** Non-research

108. Bruinsma F, Lynch B, Southey M, Giles G, Milne R. 1152Use of Facebook to recruit cohort study participants. *Int J Epidemiol*. 2021;50(Supplement_1). doi:10.1093/ije/dyab168.099

***Reason for exclusion:*** Records not available

109. Wasfi R, Stephens ZP, Sones M, et al. Recruiting participants for population health intervention research: effectiveness and costs of recruitment methods for a cohort study. *J Med Internet Res*. 2021;23(11):N.PAG-N.PAG. doi:10.2196/21142

***Reason for exclusion:*** Wrong concept

110. Dickerson DL, Parker J, Johnson CL, Brown RA, D’Amico EJ. Recruitment and retention in randomized controlled trials with urban American Indian/Alaska Native adolescents: challenges and lessons learned. *Clin Trials*. 2021;18(1):83-91. doi:10.1177/1740774520971774

***Reason for exclusion:*** Wrong concept

111. Jorrat D. Recruiting experimental subjects using WhatsApp. *J Behav Exp Econ* . 2021;90(November 2020):101644. doi:10.1016/j.socec.2020.101644

***Reason for exclusion:*** Wrong concept

112. Wentzell K, Walker HR, Hughes AS, Vessey JA. Engaging Social Media Influencers to Recruit Hard-to-Reach Populations. *Nurs Res*. 2021;70(6):455-461. doi:10.1097/NNR.0000000000000544

***Reason for exclusion:*** Wrong concept

113. Tan KR, Killela MK, Leckey J. Ethical considerations of social media to recruit caregivers of children with cancer. *Nurs Res*. 2021;70(1):67-71. doi:10.1097/NNR.0000000000000473

***Reason for exclusion:*** Wrong concept

114. Sato K, Niimi Y, Ihara R, et al. Efficacy and cost-effectiveness of promotion methods to recruit participants to an online screening registry for Alzheimer disease prevention trials: observational study. *J Med Internet Res*. 2021;23(7):e26284. Published 2021 Jul 22. doi:10.2196/26284

***Reason for exclusion:*** Wrong concept
