# Supplementary material for: Social Media Use for Research Participant Recruitment: Integrative Literature Review
Source: J Med Internet Res. 2022 Aug 4;24(8):e38015. doi: 10.2196/38015 (PMC9389385; doi:10.2196/38015)
Supplement: Multimedia Appendix 6 [file jmir_v24i8e38015_app6.docx]

**Multimedia Appendix 6: Tentative Guidelines to Assist Health Sciences Researchers in Social Media Recruitment**

The authors of this review have provided tentative guidelines to assist researchers in utilizing the social media space for recruitment. These guidelines are based on the knowledge from this synthesis and the recommendations from researchers. In addition to the recommendations regarding best practices to implement in social media recruitment presented in the main manuscript (Table 5), this guide adds information related to the cost models, recruitment and advertisement strategies, and ethical considerations.

Cost Elements to Consider in Planning Social Media Recruitment:

- In budgeting for the cost of social media recruitment, researchers should include personnel cost (research assistant, social media specialist or third-party service organization), resources (cost of advertising of social media platform, choice of cost model to use).
- Researchers can opt for either the cost-per-click model, cost-per-thousand-impressions model, cost-per-view, or cost-per-action or conversion model. These cost for each model differs and have their peculiar benefits.

Advertising /Recruitment Strategies:

- Researchers who have resources available can employ advertising and recruitment service organizations to provide support in recruiting potential participants.
- Craft advertising messages that are creative, simple, concise, and motivate participants without introducing bias to the targeted population.
- Use appealing images and consistent language through both the text caption and image.
- In crafting social media advertisement messages, researchers should develop multiple targeted themes.
- When engaging with diverse social media platforms, the advertisement design should be tailored to each platform because different platforms have specific specifications, e.g., word count and design layout (the display and design of a desktop application differs from that of a mobile application).
- For maximum reach, the advertisement should be targeted to the specific population and the geographical area.
- Some social media platforms such as Facebook, Instagram, Twitter, Craigslist, Tumblr, and Reddit offer other options such as unpaid, untargeted advertisements, and posts. Researchers can also explore these options.
- Develop multiple advertisement campaigns consisting of different themes.
- Researchers should refer to the social platform's advertising policies for details on the advertisement content.
- For multiple advertisements, researchers can rotate and alternate the advertisement campaigns between geographical locations.
- Research staff should be assigned to monitor the advertisement to determine how well an advertisement is responding to the recruitment drive. Depending on the response, advertisement campaigns can simultaneously run or be alternated.

Ethical Conduct:

- Researchers can post a secure link on social media platforms that redirects interested participants to a secure site/webpage for recruitment information. The social media platforms should be used for research advertisement purposes only.
- Researchers are responsible for the regular review of social media sites for updates on privacy settings.
- For social media postings, researchers should have a dedicated research-specific account from which all posts are made.
